# Caught in a Trap? Proteomic Analysis of Neutrophil Extracellular Traps in Rheumatoid Arthritis and Systemic Lupus Erythematosus

**DOI:** 10.3389/fimmu.2019.00423

**Published:** 2019-03-11

**Authors:** Elinor A. Chapman, Max Lyon, Deborah Simpson, David Mason, Robert J. Beynon, Robert J. Moots, Helen L. Wright

**Affiliations:** ^1^Department of Musculoskeletal Biology I, Institute of Ageing and Chronic Disease, University of Liverpool, Liverpool, United Kingdom; ^2^Department of Biochemistry, Institute of Integrative Biology, University of Liverpool, Liverpool, United Kingdom; ^3^Centre for Proteome Research, University of Liverpool, Liverpool, United Kingdom; ^4^Centre for Cell Imaging, University of Liverpool, Liverpool, United Kingdom; ^5^University of Liverpool and Aintree University Hospital, Members of Liverpool Health Partners, Liverpool, United Kingdom

**Keywords:** neutrophil, neutrophil extracellular trap, NET, rheumatoid arthritis, systemic lupus erythematosus, citrullinated, histones

## Abstract

Neutrophil Extracellular Traps (NETs) are implicated in the development of auto-immunity in diseases such as rheumatoid arthritis (RA) and systemic lupus erythematosus (SLE) through the externalization of intracellular neoepitopes e.g., dsDNA and nuclear proteins in SLE and citrullinated peptides in RA. The aim of this work was to use quantitative proteomics to identify and measure NET proteins produced by neutrophils from healthy controls, and from patients with RA and SLE to determine if NETs can be differentially-generated to expose different sets of neoepitopes. Ultra-pure neutrophils (>99%) from healthy individuals (*n* = 3) and patients with RA or SLE (*n* = 6 each) were incubated ± PMA (50 nM, PKC super-activator) or A23187 (3.8 μM, calcium ionophore) for 4 h. NETs were liberated by nuclease digestion and concentrated onto Strataclean beads prior to on-bead digestion with trypsin. Data-dependent LC-MS/MS analyses were conducted on a QExactive HF quadrupole-Orbitrap mass spectrometer, and label-free protein quantification was carried out using Progenesis QI. PMA-induced NETs were decorated with annexins, azurocidin and histone H3, whereas A23187-induced NETs were decorated with granule proteins including CAMP/LL37, CRISP3, lipocalin and MMP8, histones H1.0, H1.4, and H1.5, interleukin-8, protein-arginine deiminase-4 (PADI4), and α-enolase. Four proteins were significantly different between PMA-NETs from RA and SLE neutrophils (*p* < 0.05): RNASE2 was higher in RA, whereas MPO, leukocyte elastase inhibitor and thymidine phosphorylase were higher in SLE. For A23187-NETs, six NET proteins were higher in RA (*p* < 0.05), including CAMP/LL37, CRISP3, interleukin-8, MMP8; Thirteen proteins were higher in SLE, including histones H1.0, H2B, and H4. This work provides the first, direct comparison of NOX2-dependent (PMA) and NOX2-independent (A23187) NETs using quantitative proteomics, and the first direct comparison of RA and SLE NETs using quantitative proteomics. We show that it is the nature of the stimulant rather than neutrophil physiology that determines NET protein profiles in disease, since stimulation of NETosis in either a NOX2-dependent or a NOX2-independent manner generates broadly similar NET proteins irrespective of the disease background. We also use our proteomics pipeline to identify an extensive range of post-translationally modified proteins in RA and SLE, including histones and granule proteins, many of which are known targets of auto-antibodies in each disease.

## Introduction

Neutrophil Extracellular Traps (NETs) are chromatin-derived extracellular “spider webs” that are expelled from neutrophils in response to infectious or inflammatory stimuli (1). They were first described as an alternative defense mechanism by which neutrophils trap and possibly kill microbes (1), with subsequent studies confirming this (2–6). Microbes including bacteria, fungi and viruses have varying susceptibility to NETs, either being trapped and/or killed, or having their growth inhibited (7). NET DNA structures are decorated with histones, myeloperoxidase (MPO), and other antimicrobial proteins such as neutrophil elastase. Some NET proteins, including histones, may be post-translationally modified with methylated, acetylated, and/or citrullinated residues (8–12). Many inducers of NET release (NETosis) have been identified, including physiological and non-physiological molecules, and micro-organisms including gram-positive and gram–negative bacteria and fungi. The most potent and commonly used non-physiological inducer of NETosis *in vitro* is phorbol 12-myristate 13-acetate (PMA) (13–15), a super-activator of protein kinase C (PKC). Calcium ionophores such as ionomycin and A23187 also induce the release of NETs containing, in particular, citrullinated histones (9–12, 16, 17). Many, more physiologically relevant inducers of NETs have been reported, including N-Formylmethionyl-leucyl-phenylalanine (fMLP), interleukin-8 (IL-8), lipopolysaccharide (LPS), Platelet toll-like receptor (TLR)-4, Nitric Oxide, and TNFα (18–21), although IL-8-induced NET formation may be sensitive to cell culture conditions(22).

In many cases, activation of the NADPH-oxidase (NOX2) and generation of reactive oxygen species (ROS) is required for NET formation (NOX2-dependent NETosis). ROS increase membrane permeability, leading to the release of neutrophil elastase into the nucleus, which first degrades linker H1 histones followed by core histones driving chromatin decondensation, a process enhanced by MPO (23). ROS also promote the morphological changes that occur during NETosis (24), inhibit apoptosis, and induce autophagy (23), with the level of intracellular ROS determining whether the autophagy reaction leads to NETosis (24). Many agonists, including PMA, induce NOX2-dependent NET production (15, 24), which is regulated by the Raf-MEK-ERK pathway (13, 25). There are also conflicting reports about the involvement of RIPK1, RIPK3, and MLKL signaling pathways in PMA-induced NETosis (26, 27). NETosis induced by calcium ionophores such as A23187 and activated platelets occurs in a different manner, independent of NOX2 activity and thus is often referred to as NOX2-independent NET formation (17, 28). NOX2-independent NETosis is dependent on intracellular calcium and activation of peptidylarginine deiminase (PAD) enzymes leading to hypercitrullination of histones (9, 17). Recent work suggests that activation of PAD induces citrullination of p47^phox^ and p67^phox^ proteins, preventing assembly of active NOX2 and production of NOX2-dependent ROS (29). NOX2-independent NETosis relies upon the production of mitochondrial ROS (mtROS) and activation of the calcium-activated small conductance potassium (SK) channel member SK3 (17). Elastase does not seem to be required for NOX2-independent NETosis (30), and neither are F-actin or histones cleaved (23, 31).

Neutrophils are implicated in the pathogenesis of several inflammatory diseases including rheumatoid arthritis (RA) and systemic lupus erythematosus (SLE) (32–36). Both diseases are characterized by a dysregulation of neutrophil activation, including cytokine and ROS production, gene expression and apoptosis (35, 37, 38). RA is a chronic, inflammatory disease affecting the joints, characterized by inflammation of synovial tissues and irreversible damage to cartilage and bone within synovial joints. In RA, neutrophil apoptosis is delayed, particularly within RA joints (39, 40), and activated neutrophils within synovial tissues and synovial fluid release cytotoxic proteases that damage cartilage. SLE is a heterogeneous auto-immune condition with multiple organ involvement, including the skin, kidneys, cardiovascular system, central nervous system, and joints (41, 42). In SLE, neutrophil apoptosis is enhanced leading to an increase in apoptotic burden associated with development of anti-nuclear auto-antibodies (43, 44). Neutrophils are implicated in the development of auto-immunity in both diseases through the production of NETs and externalization of intracellular neoepitopes e.g., dsDNA and nuclear proteins in SLE and citrullinated peptides in RA (8, 21, 45–50).

Increased spontaneous NETosis has been observed in several autoimmune conditions (51–53), including SLE (45, 46) and RA (21), and sera from SLE and RA patients activate neutrophils and cross-react with NET components (8, 21, 47–50). Serum levels of MPO-DNA complexes (NET remnants) are elevated in patients with RA, SLE, primary Sjögren's syndrome, dermatomyositis, and ankylosing spondylitis (54, 55). Analysis of NET remnants in the serum of patients with RA and SLE suggests NETs produced in both diseases originate via NOX2-independent NETosis (55). In RA, MPO-DNA levels are associated with increased neutrophil counts and positivity for rheumatoid factor (RF) and anti-citrullinated protein/peptide antibodies (ACPA) (54, 56). NETs are implicated in the activation of plasmacytoid dendritic cells in SLE (57), and may induce damage to endothelial tissues and organs (44, 45, 58, 59). NETs and NET-derived proteases activate coagulation, and provide a scaffolding for clot assembly, often associated with SLE (60, 61). NETs produced by both SLE neutrophils and SLE low-density granulocytes (LDGs) damage endothelial cells (44, 45, 58).

Previous proteomics studies have identified a number of proteins decorating NETs produced by both healthy and inflammatory neutrophils, including granule proteins (elastase, MPO, azurocidin, lactoferrin, gelatinase), histones (H1, H2A, H2B, H3, H4) and S100 family proteins (S100A8, S100A9, S100A10) (21, 62–64). However, these reports were descriptive and only semi-quantitative. The purpose of our study was to carry out the first fully quantitative proteomics analysis of NETs produced by RA and SLE neutrophils in response to NOX2-dependent and NOX2-independent activation, and to determine whether the protein composition of NETs is agonist and/or disease specific. We describe a comprehensive and quantitative proteomics approach to examining the composition of PMA- and A23187-induced NETs from ultrapure RA and SLE neutrophils, which has identified over 450 NET proteins. We have also analyzed these data to identify NET proteins for post-translational modifications on peptides, many of which correspond to known auto-antibody species in RA and SLE. We show that the mode of stimulation of NETosis (NOX2-dependent or NOX2-independent) generates broadly similar profiles of NET proteins irrespective of the disease background, and that it is the nature of the stimulant rather than the neutrophil physiology that determines NET protein profiles in disease. However, we also describe a number of proteins that are expressed at significantly higher levels on NETs in RA or SLE and which may have important contributions to disease pathology in these two very different inflammatory conditions.

## Methods

### Patients

All patients fulfilled ACR criteria for RA or SLE (65, 66) and were recruited from clinics at University Hospital Aintree. Clinical demographics for patients included in the study are shown in Table 1. All patients had active disease as determined by a clinician at the time of sample collection.

**Table 1:** Patient clinical demographics.

	**RA**	**SLE**
*N*	6	6
Age (years)^a, b^	61.72 (22.1–77.23)	38.3 (23.1–62.7)
Disease duration (years)^a^	1.8 (0–5.2)	4.2 (0–9.7)
Gender F/M	3/3	5/1
ACPA pos/neg	5/1	0
Anti-dsDNA pos/neg	0	6/0
Disease activity score^a^	4.04 (3.26–5.21)^c^	6 (0–12)^a,d^
**CURRENT DRUG TREATMENTS**		
– Methotrexate	4	1
– Sulphasalazine	1	0
– Hydroxychloriquine	2	4
– Prednisolone	0	4
– Belimumab	0	2
– Mycophenolate mofetil	0	2

a *Mean (range)*.

b *T-test (p = 0.05)*.

c *DAS28*.

d *SLEDAI scores available for 2/6 patients*.

### Materials

HetaSep solution and EasySep Human Neutrophil enrichment kit were from StemCell (Cambridge, UK); Ficoll-Paque was from GE Healthcare (Little Chalfont, UK); RPMI 1640 media plus without phenol red, L-glutamine, 25 mM HEPES and Hanks Balanced Salt Solution (HBSS), Annexin V-FITC, anti-rabbit AlexaFluor488, and anti-mouse AlexaFluor647 were from Life Technologies (Paisley, UK); Rapid Romanowsky stain was from TCS Biosciences (Botolph Claydon, UK); anti-CD16-FITC was from BD Biosciences (Oxford, UK); Propidium Iodide, A23187, phorbol 12-myristate 13-acetate (PMA), R848, LPS, DAPI, Mowiol 4–88, micrococcal nuclease, poly-L-lysine, human AB serum and PhastGel® Blue R Pre-measured tablets were from Sigma (Gillingham, UK); rabbit anti-neutrophil elastase antibody, mouse anti-myeloperoxidase antibody, rabbit anti-CRISP3 antibody, rabbit anti-thymidine phosphorylase antibody, rabbit anti-histone H2B, and mouse anti-PADI4 antibody were from Abcam (Cambridge, UK); goat anti-MMP8 antibody, mouse anti-CAMP (LL37) antibody, mouse anti-LCN1 antibody, and mouse anti-histone H1.0 antibody were from Biotechne (Abingdon, UK); Quantifluor dsDNA kit was from Promega (Southampton, UK); 96-well black plates, lithium-heparin vacutainers and Z-serum clot activator vacutainers were from Greiner (Stonehouse, UK); SYTOX® Green Nucleic Acid Stain and 0.5 M UltraPure EDTA pH8.0 was from ThermoFisher (Loughborough, UK); Strataclean beads were from Agilent (Cheadle, UK), cover slips were from Fischer Scientific (Loughborough, UK); MALP-2 was from Enzo Life Sciences (Exeter, UK); TNFα was from Merck (Watford, UK).

### Isolation of Neutrophils

Blood was collected into lithium-heparin vacutainers. Ultra-pure neutrophils (>99%) were isolated from erythrocyte-depleted (HetaSep) whole blood as previously described using Ficoll-Paque followed by the EasySep Human Neutrophil enrichment kit (14, 67). Highly-pure (>99%) neutrophils were briefly centrifuged and resuspended in RPMI 1640 media containing L-glutamine, plus 25 mM HEPES, to a concentration of 5 × 10^6^/mL. Neutrophil purity was confirmed by anti-CD16-FITC staining and morphology. Cytospins were prepared by centrifugation of 10^5^ cells (in PBS with 10 mM EDTA) onto a glass slide at 30 g for 5 min using a Shandon 3 cytospin and immediately stained with Rapid Romanowsky stain.

### Isolation of Patient Serum and Synovial Fluid

RA SF from knee joints was aspirated into heparinized tubes and processed within 1 h. Aliquots of whole SF were centrifuged at >2,000 g for 5 min and cell-free SF was decanted and frozen at −80°C. RA serum was obtained by centrifuging blood drawn into *Z* serum clot activator tubes at 1,500 g for 10 min at RT, before freezing at −80°C.

### Measurement of Apoptosis

Neutrophils were incubated at 37°C in 5% CO_2_ in RPMI 1640 in the absence or presence of human AB serum (0–2%) for 5 h. Following incubation, 2.5 × 10^4^ cells were diluted in 50 μL of HBSS containing 0.5 μL Annexin V-FITC, and incubated in the dark at room temperature for 15 min. The total volume was then made up to 500 μL with HBSS containing propidium-iodide (PI, 1 μg/mL) before analysis by flow cytometry (>5,000 events analyzed) using a Guava EasyCyte flow cytometer.

### Measurement of ROS Production

Neutrophils (5 × 10^6^/mL) were incubated with PMA (50 nM), A23187 (3.8 μM) or vehicle control (DMSO). Luminol-enhanced chemiluminescence (luminol, 10 μM) was measured continuously for 30 min using a Tecan plate reader at 37°C.

### Visualization of NET Production by Immunofluorescence

Neutrophils were seeded (at 2 × 10^5^ cells/500 μL) in RPMI media plus 2% AB serum in duplicate wells (with or without a glass coverslip in the well) of a 24-well plate. Cells were allowed to adhere for 1 h prior to stimulation with PMA (50 nM), A23187 (3.8 μM) or vehicle control (DMSO). In preliminary experiments neutrophils were also stimulated with TNFα (100 ng/mL), LPS (100 ng/mL), MALP-2 (100 ng/mL), R848 (5 μM), soluble immune complexes (SIC, 10%), insoluble immune complexes (IIC, 10%) RA synovial fluid (SF, 10%), or autologous serum (10%). Immune complexes were prepared as previously described (68). Cells were incubated for a further 4 h to allow for NET production. Cells adhered to coverslips were fixed with 4% paraformaldehyde prior to immunofluorescent staining. Briefly, coverslips were removed from the plate and washed with PBS, permeabilised with 0.05% Tween 20 in TBS, fixed with TBS (2% BSA), and then stained for 30 min on drops of TBS (2% BSA) on parafilm stretched across a clean 24-well plate. Primary antibodies used were: rabbit anti-neutrophil elastase (1:200), mouse anti-myeloperoxidase (1:1,000), rabbit anti-citrullinated histone H3 (1:250), rabbit anti-CRISP3 (1:100), rabbit anti-thymidine phosphorylase (1:200), rabbit anti-histone H2B (1:100), mouse anti-PADI4 (1:100), goat anti-MMP8 (1:15), mouse anti-CAMP(LL37) (1:10), mouse anti-LCN1 (1:200), and mouse anti-histone H1.0 (1:200). Coverslips were washed three times with TBS prior to secondary antibody staining (anti-rabbit AlexaFluor488, 1:2,000, anti-mouse AlexaFluor647, 1:2,000, or anti-goat AlexaFluor647, 1:2,000) in TBS (+2% BSA) for 30 min. Coverslips were washed prior to staining with DAPI (1 μg/mL) for 3 min. Coverslips were washed a further 3 times and mounted onto glass slides using Mowiol 4–88. Slides were imaged on either an Epifluorescent microscope (Zeiss) or a confocal laser-scanning microscope (Leica DM2500) using a 20X, 40X, or 100X objective.

### Quantitative Analysis of Immunofluorescent NET Images

Images were analyzed using Fiji (69) with equal color balance. The DAPI channel of one image from each condition was used to train a machine learning pixel classifier in Ilastik v1.3.0 (70) to recognize three categories: background, compact nuclei, and NETs. Subsequently, all images in the dataset were processed to produce a simple segmentation count mask output. A Fiji (69) script was used to measure the area occupied by each label (available at https://bitbucket.org/snippets/davemason/5edXBB).

### Quantitative Measurement of DNA Released During NETosis

Two assays were used to measure the release of DNA during NETosis. In parallel experiments to those described above, neutrophils were seeded (5 × 10^5^ cells/500 μL) in RPMI media plus 2% AB serum in a 24-well plate. Cells were allowed to adhere for 1 h prior to stimulation with PMA (50 nM), A23187 (3.8 μM), or vehicle control (DMSO). Cells were incubated for a further 4 h to allow for NET production. Following a total of 5 h incubation, 5 μM CaCl_2_ was added to culture supernatant followed by 500mU micrococcal nuclease and incubated for 10 min at 37°C. The nuclease reaction was stopped by the addition of 5 μL EDTA (0.5 M). Culture supernatants were removed from each well, centrifuged at 200 g for 5 min to remove cellular debris, and decanted into clean tubes prior to freezing at −80°C. DNA content of each supernatant was measured using the Quantifluor dsDNA kit in black 96-well plates using serially diluted lambda DNA as a calibration standard (0–2,000 ng/mL). Measurement was carried out at 485 nm excitation/535 nm emission on a Tecan plate reader. In addition, purified neutrophils (2 × 10^5^ cells/200 μL) were seeded in 96-well black plates in the presence of 5 μM SYTOX Green Nucleic Acid Stain, a non-cell-permeable DNA binding dye. Cells were then stimulated as described above and incubated at 37°C. Plates were read every 30 min for 4 h on a Tecan plate reader. SYTOX green excites at 480/490 nm and emits at 520 nm [adapted from (16)].

### Preparation of NET Samples for Proteomics Analysis

Neutrophils were plated at 1.7 × 10^6^ cells/mL in RPMI without phenol red, and supplemented with L-glutamine, 25 mM HEPES plus 2% AB serum in 12-well plates at 37°C in a 5% CO_2_ incubator. Cells were allowed to settle for 1 h, stimulated as described above and incubated for a further 4 h. After 4 h, the supernatant was removed and the cells were washed twice for 10 min with RPMI media (with no serum). Finally 1 mL RPMI media containing 1 μM CaCl_2_ and 50 mU micrococcal nuclease was added and incubated for 20 min to digest NET DNA. Five micromoles EDTA was used to stop the reaction. Digested NET material was removed by gently tilting the plate and removing the supernatant into a 1.5 mL Eppendorf tube. The supernatant was centrifuged at 400 g for 5 min at 4°C, carefully transferred to a clean tube and the centrifuged at 16,000 g for 5 min at 4°C. We have previously used protein absorption onto Strataclean beads to concentrate samples prior to proteomics (71). The supernatant fraction was carefully transferred to a clean tube, 10 μL Strataclean beads were added and the suspension was vortexed for 1 min at room temperature. The beads were then washed twice in 1 mL ice cold-PBS, by centrifuging at 2,000 g for 2 min at 4°C, before freezing at −80°C for later proteomics analysis. This protocol was developed and adapted from previous studies (62, 63).

The beads were re-suspended in 80 μL of 25 mM ammonium bicarbonate (ambic) and 5 μL of 1%(w/v) Rapigest (Waters) in 25 mM ambic, and the samples heated at 80°C for 10 min with shaking. Samples were reduced by the addition of 5 μL of 60 mM DTT in 25 mM ambic and heated at 60°C for 10 min. Samples were cooled and 5 μL of 180 mM iodoacetamide in 25 mM ambic was added, and samples incubated at room temp for 30 min in the dark. Trypsin (Promega Gold sequencing grade) (1 μg) was added and the samples incubated at 37°C overnight on a rotary mixer. The following day the digests were acidified (to remove Rapigest surfactant) by the addition of 1%(v/v) TFA (acidity checked using pH paper) and incubated at 37°C for 45 min. Samples were then centrifuged at 17,000 g for 30 min and the clarified supernatants transferred to 0.5 mL low-bind tubes. Samples were centrifuged for a further 30 min and 10 μL transferred to total recovery vials for LC-HRMS analysis.

For visualization of NET proteins by SDS-PAGE, Strataclean beads were first boiled for 5 min in 25 μL Laemmli buffer to liberate proteins. The samples were briefly centrifuged to sediment the Strataclean beads before loading the total volume of sample on a 10% SDS-PAGE and running at 100 V for 1 h. Gels were stained with PhastGel® Blue. One RA PMA sample was found to contain insufficient protein and was excluded from the analysis.

### LC-MS/MS Analysis

Data-dependent LC-MS/MS analyses were conducted on a QExactive HF quadrupole-Orbitrap mass spectrometer coupled to a Dionex Ultimate 3000 RSLC nano-liquid chromatograph (Hemel Hempstead, UK). Sample digest (4–8 μL) was loaded onto a trapping column (Acclaim PepMap 100 C18, 75 μm × 2 cm, 3 μm packing material, 100 Å) using a loading buffer of 0.1% (v/v) TFA, 2% (v/v) acetonitrile in water for 7 min at a flow rate of 9 μL/min. The trapping column was then set in-line with an analytical column (EASY-Spray PepMap RSLC C18, 75 μm × 50 cm, 2 μm packing material, 100 Å) and the peptides eluted using a linear gradient of 96.2% A (0.1% [v/v] formic acid):3.8% B [0.1% [v/v] formic acid in water:acetonitrile [80:20]] to 50% A:50% B over 30 min at a flow rate of 300 nL/min, followed by washing at 1% A:99% B for 5 min and re-equilibration of the column to starting conditions. The column was maintained at 40°C, and the effluent introduced directly into the integrated nano-electrospray ionization source operating in positive ion mode. The mass spectrometer was operated in DDA mode with survey scans between *m*/*z* 350–2,000 acquired at a mass resolution of 60,000 (FWHM) at *m*/*z* 200. The maximum injection time was 100 ms, and the automatic gain control was set to 3e6. The 10 most intense precursor ions with charges states of between 2+ and 5+ were selected for MS/MS with an isolation window of 2 *m*/*z* units. The maximum injection time was 100 ms, and the automatic gain control was set to 1e5. Fragmentation of the peptides was by higher-energy collisional dissociation using a normalized collision energy of 29%. Dynamic exclusion of *m*/*z* values to prevent repeated fragmentation of the same peptide was used with an exclusion time of 20 s.

### Data Analysis

Acquired MS data was searched in Mascot (v2.6.2, Matrix Science, London, UK) against the reviewed entries of the reference proteome set of *H. sapiens* from Uniprot (20,328 sequences) using a peptide mass tolerance of 10 ppm and a fragment ion tolerance of 0.01Da. Carbamidomethyl cysteine and oxidation of methionine were selected as fixed and variable modifications, respectively. Additional searches were conducted using PEAKs (v8.5, Bioinformatics Solutions Inc., Waterloo, Ontario, Canada) (72) to identify protein citrullination, acetylation, and methylation, which in addition to methionine oxidation were specified as variable modifications. A precursor mass tolerance of 10 ppm and a fragment ion mass tolerance of 0.01 Da was used. A peptide false discovery rate of 1% was set. Label-free protein quantification was performed using Progenesis QI for Proteomics v.2.0 (Waters Ltd., Newcastle-upon-Tyne, UK). Samples were automatically aligned according to retention time. Default peak picking parameters were applied and peptides with charges between 2+ and 7+ were retained. Database searching was performed using Mascot. A Mascot Generic File, created by Progenesis, was searched against the UniProt human reviewed database with modifications and mass tolerances as specified above. Search results were filtered to obtain a peptide false discovery rate of 1% before importing into back into Progenesis. Protein quantification was based on averaging the individual abundances for every unique peptide for each protein and comparing the relative abundance across sample runs and between experimental groups. The mass spectrometry proteomics data have been deposited to the ProteomeXchange Consortium via the PRIDE (73) partner repository with the dataset identifiers PXD011796 and doi: 10.6019/PXD011796.

In addition to analyzing the NET proteins using this methodology, we also measured the proteins present in a cell-free protein preparation (*n* = 4) from RPMI plus 2% AB serum to identify those proteins present in the culture media (*n* = 243 proteins, Supplementary Table 1). Any proteins detected in the NET samples that corresponded with this list were excluded from our analyses.

### Statistical Analysis

Experimental data were tested for normality using the Kolmogorov-Smirnov test, and then analyzed using Student's *t*-test or Mann-Whitney *U*-test as appropriate in GraphPad Prism (version 4). Proteomics data was analyzed using ANOVA in Progenesis and Pearson's correlation in R. Bespoke R scripts were used to carry out principal component analysis. Heatmaps were produced using MeV (v4) (74). Gene Ontology analysis was carried out using DAVID (v6.8) (75, 76) and is reported as a Benjamini-adjusted *p*-value (False Discovery Rate, FDR).

## Results

### Optimization of NET Protocol for Proteomics

**Figure 1:**
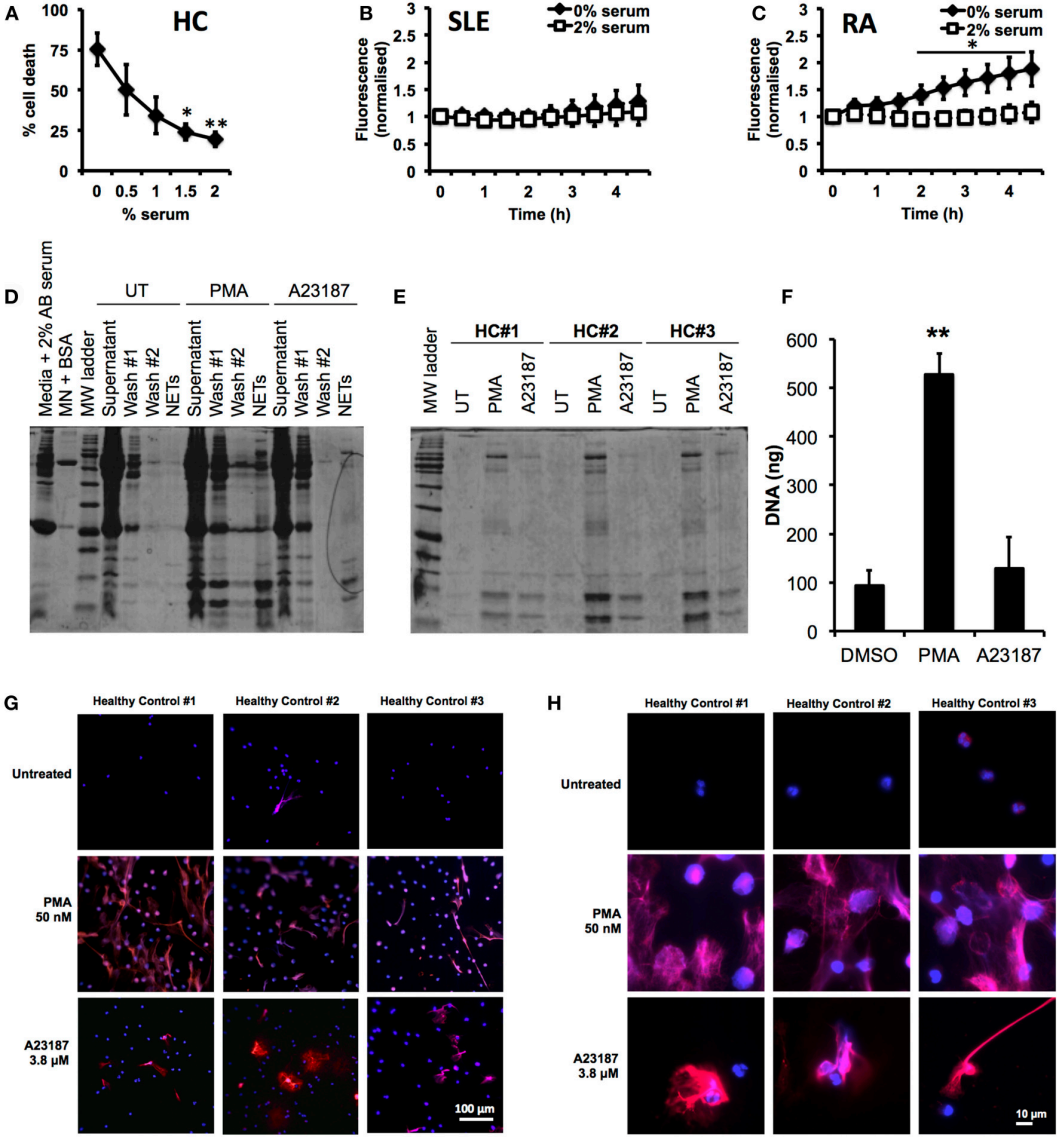
Optimization of incubation conditions for NET proteomics. **(A)** Titration of human AB serum (0–2%) in culture media significantly inhibited cell death detected by annexin V/PI in healthy neutrophils (*n* = 3 ^*^p < 0.05, ^**^p < 0.01). Supplementation of 2% AB inhibited externalization of DNA detected by SYTOX green assay in SLE **(B)** and RA **(C)** neutrophils (*n* = 6, ^*^p < 0.05). **(D)** Optimization of wash steps to remove media containing serum proteins from NET preparations prior to proteomic analysis. **(E)** SDS-PAGE gel of NET proteins prepared from three healthy donors in response to PMA (50 nM) and A23187 (3.8 μM). **(F)** Detection of DNA in culture media of neutrophils treated with A23187, PMA, or DMSO (vehicle control). Significantly more NETosis was observed in response to PMA (*n* = 3, ^**^p < 0.01). This was confirmed by immunofluorescent staining for DNA (DAPI,blue) and myeloperoxidase (red). Images taken on an Epifluorescent microscope (Zeiss) at X20 **(G)** and X100 **(H)** magnification.

First we optimized our neutrophil culture conditions, as previous studies have demonstrated that NETosis is highly sensitive to pH and CO_2_ concentration (77), as well as serum concentration (15, 22, 78). In particular we wished to minimize the amount of serum used in our incubations, to prevent excessive carry-through of serum proteins into the proteomics analysis pipeline. Several recent studies (8, 21, 62, 63) have excluded serum from NETosis experiments; however we found that serum-free media caused high levels of cell death after 5 h (75.3 ± 10.3%, *n* = 3) and, in particular PI positive, necrotic cells (14.0 ± 5.8%, *n* = 3) in healthy, untreated neutrophils. Titration of serum in culture medium (0–2%) significantly decreased the amount of cell death after 5 h incubation (Figure 1A, 2% serum, 19.5 ± 4.5%, *p* < 0.01). The effect of serum supplementation on NETosis was confirmed in RA and SLE neutrophils using the SYTOX Green assay. Serum-free media increased the amount of DNA released by neutrophils over 4.5 h (Figures 1B,C). This was markedly increased in RA neutrophils, where the absence of serum caused a 2-fold increase in DNA release by 4.5 h (Figure 1C, *n* = 6, *p* < 0.05). These results mirrored the visible production of NETs in the absence or presence of up to 2% serum (Supplementary Figure 1). Based on these data we decided to use 2% AB serum in our experiments.

Our next step was to minimize the carry-through of serum proteins to our proteomics pipeline. We found that inclusion of two gentle, 10 min wash steps significantly removed the amount of protein visible on a PhastGel® Blue stained SDS-PAGE gels (Figure 1D). We also titrated the concentration of micrococcal nuclease used during NET digestion, determining that 50 mU was sufficient to liberate NETs, as shown by the Quantifluor assay, Coomassie gel and immuno-fluorescent staining (Supplementary Figure 2). We observed differences in the quantity of NET proteins produced in response to PMA or A23187 by SDS-PAGE gel analysis (Figure 1E). These observations matched both measurements of NET DNA in culture supernatants, and immunofluorescent staining of NETs, prepared in parallel experiments (Figures 1F,G). Not only did A23187 produce fewer NETs, but also the NETs produced in response to A23187 and PMA were visually very different (Figures 1G,H). Whilst PMA-induced NETs tended to emanate from a neutrophil which had swollen to almost double its size before bursting, A23187-induced NETs appeared to emanate from cells which had condensed and ejected their DNA, while still partially retaining their nuclear structure (Figures 1G,H).

**Figure 2:**
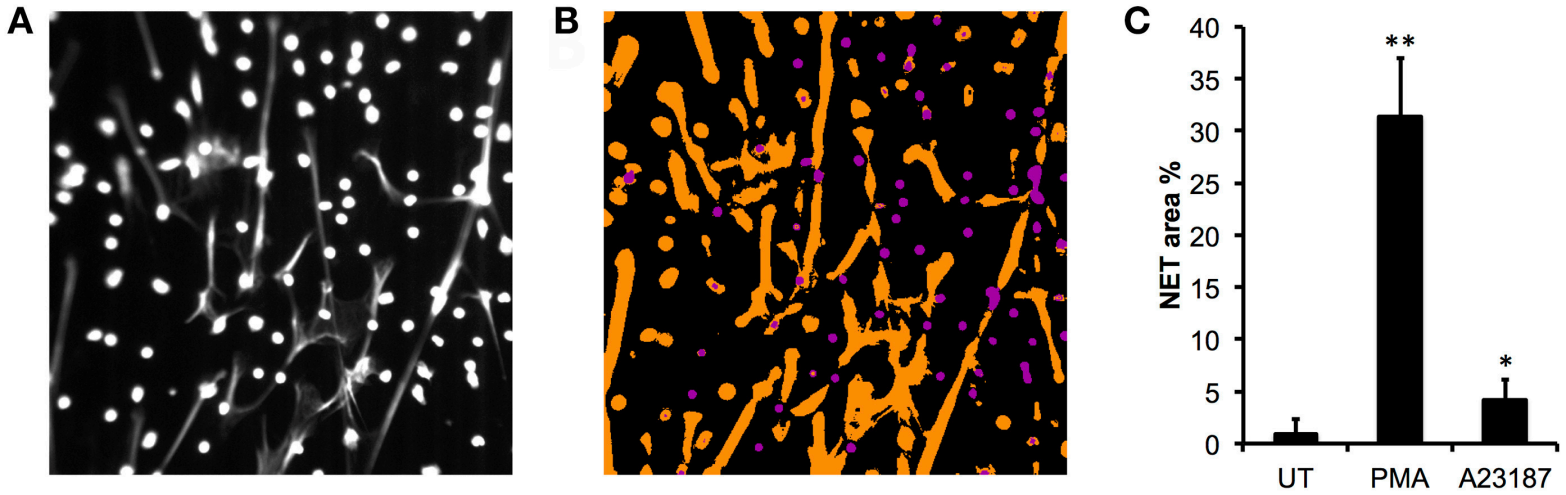
Quantitative analysis of NET images using Ilastik. **(A)** Representative image of DAPI stained neutrophils stimulated with PMA, is shown with contrast enhanced to ~50% saturation to more clearly show the NETs. Image taken on an Epifluorescent microscope (Zeiss). **(B)** Ilastik machine learning pixel classifier recognizes three categories: background (black), compact nuclei (purple) and NETs (orange). **(C)** Quantification of NETs from healthy control neutrophils in response to PMA (50 nM) and A23187 (3.8 μM) using Ilastik pixel classifier (*n* = 3, ^*^p < 0.05, ^**^p < 0.01, Student's *t*-test).

We validated the quantification of externalized DNA in culture supernatants by analyzing the immunofluorescent images. Characterization of the NET phenotype is a non-trivial problem given the high dynamic range of intensities involved and subtle changes in intensity distribution that occur during the process. To alleviate these issues, we turned to machine learning to more robustly characterize condensed nuclei and NETs (70). After training the model on a subset of the data, all images in the dataset were processed to produce a simple segmentation count mask output (Figures 2A,B). Quantification of the % NETs in each image using the Illastik pixel classifier (Figure 2C) showed good correlation with the picogreen assay of micrococcal nuclease-digested supernatants from parallel experiments with the same donor neutrophils (Figure 1F). Interestingly, when we measured NET material produced in response to A23187 using the picogreen assay (Figure 1F), this was not significantly different from untreated neutrophils despite visible NETs on coverslips (Figure 1G). However, NET production in response to A23187 was significant based on the immunofluorescent analysis (Figure 2C, *p* < 0.05).

### Proteomic Analysis of NETs Produced by Healthy Neutrophils

**Figure 3:**
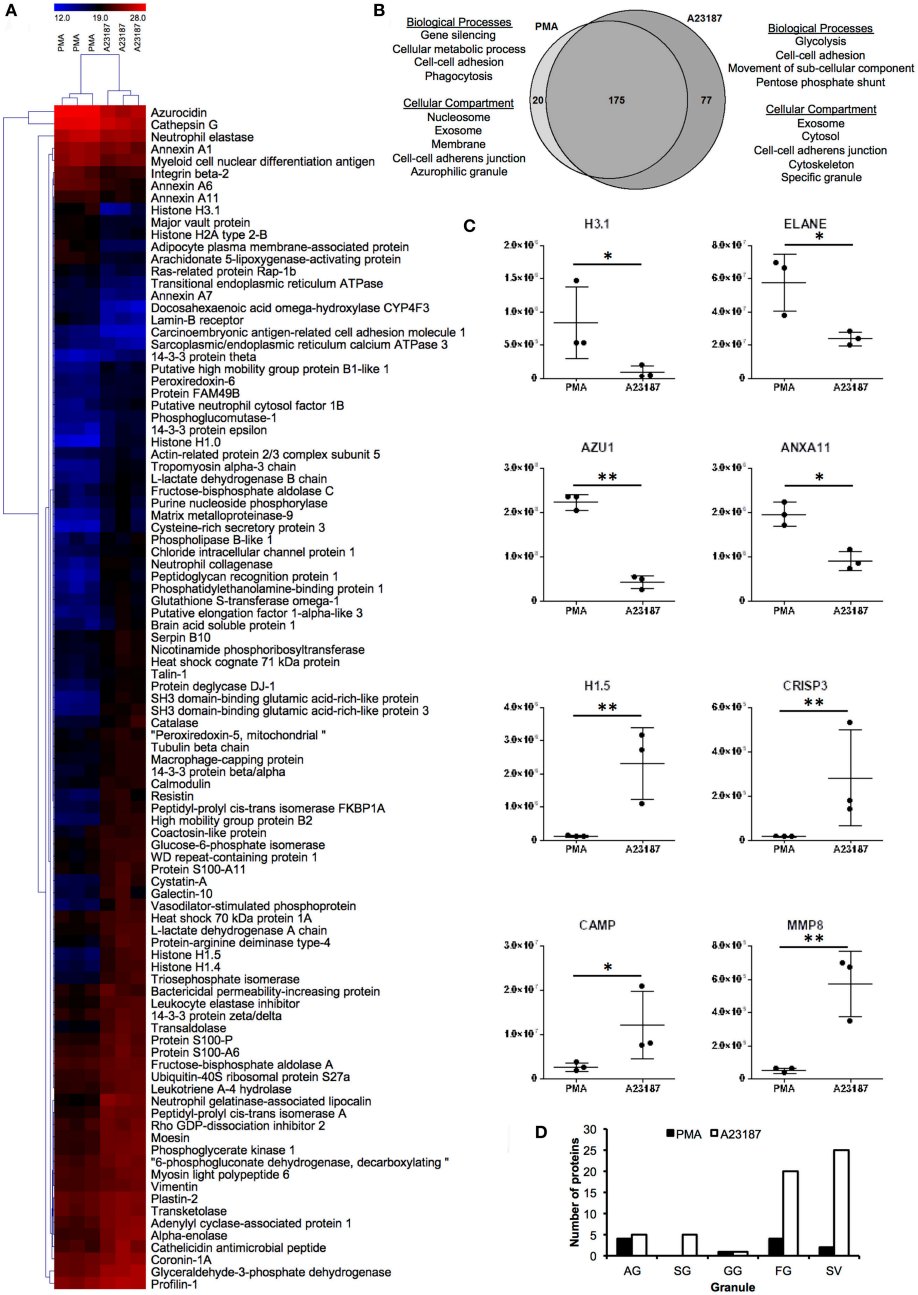
Analysis of NET proteins released in response to PMA and A23187 by healthy control neutrophils. Analysis of proteins significantly different (FDR < 0.05) between PMA- and A23187-induced NETs using **(A)** hierarchical clustering and **(B)** gene ontology. **(C)** Proteins significantly elevated in PMA-induced NETs included histone H3.1, ELANE, AZU1 ANXA11, whereas in A23187-induced NETs histone H1.5, CRISP3, CAMP, and MMP8 were significantly elevated (^*^FDR < 0.05, ^**^FDR < 0.01). Y-axis represents normalized protein abundance. **(D)** Analysis of the distribution of neutrophil granule proteins revealed differences between the source of NET proteins in response to PMA and A23187 (AG, azurophilic granule; SG, specific granule; GG, gelatinase granule; FG, ficolin-1-rich granule; SV, secretory vesicles). Shading in **(A)** relates to log2 protein abundance: blue, low; black, median; red, high.

Proteomics analysis of NETs produced by healthy control neutrophils in response to PMA (50 nM) and A23187 (3.8 μM) identified a total of 272 proteins with a minimum of 1 peptide. Filtering results to include proteins with *n* ≥ 2 peptides decreased this number to 197 (Supplementary Table 2). Of these, 97 proteins were present at significantly different amounts between the two conditions; 20 were higher in PMA-stimulated NETs and 77 were higher in A23187-stimulated NETs (Figures 3A,B, Supplementary Table 3, FDR < 0.05, fold-change ≥2, minimum 2 peptides). Gene Ontology analysis revealed that NET proteins externalized in response to PMA were associated with biological processes including: gene silencing, cellular metabolic processes, cell-cell adhesion and phagocytosis, whereas NET proteins externalized in response to A23187 were associated with glycolysis, cell-cell adhesion, movement of sub-cellular components and the pentose phosphate shunt (Figure 3B, FDR < 0.01). PMA-induced NET proteins were associated with cellular compartments including the nucleosome, exosomes, the membrane, cell-cell adherens junctions and azurophilic granules whereas A23187-induced NET proteins were associated with exosomes, the cytosol, cell-cell adherens junctions, the cytoskeleton, and specific granules (Figure 3B, FDR < 0.01). Histone 1 family proteins (H1.0, H1.4, H1.5) were significantly higher in A23187-induced NETs, whereas histone H2A and histone H3.1 were higher in PMA-induced NETs (Figure 3C, FDR < 0.05). Analysis of the distribution of neutrophil granule proteins, based on previously published proteomics analysis of sub-cellular neutrophil fractionations (79), revealed differences in types of granule proteins externalized on NETs, with azurophilic granule proteins cathepsin G (CTSG), neutrophil elastase (ELANE), and azurocidin (AZU1/CAP7) higher in PMA-induced NETs, and specific granule proteins cysteine-rich secretory protein 3 (CRISP3), cathelicidin anti-microbial peptide (CAMP/LL37), matrix metalloproteinases MMP8 and MMP9 higher in A23187-induced NETs (Figure 3C, FDR < 0.05). NETs stimulated by A23187 were also enriched with proteins from ficolin-1-rich granules (FG) and secretory vesicles (SV) (Figure 3D). Protein-arginine deiminase 4 (PADI4) was significantly higher in A23187-stimulated NETs (FDR = 0.012).

### Proteomics Analysis of NETs Produced by Neutrophils From RA and SLE Patients

**Figure 4:**
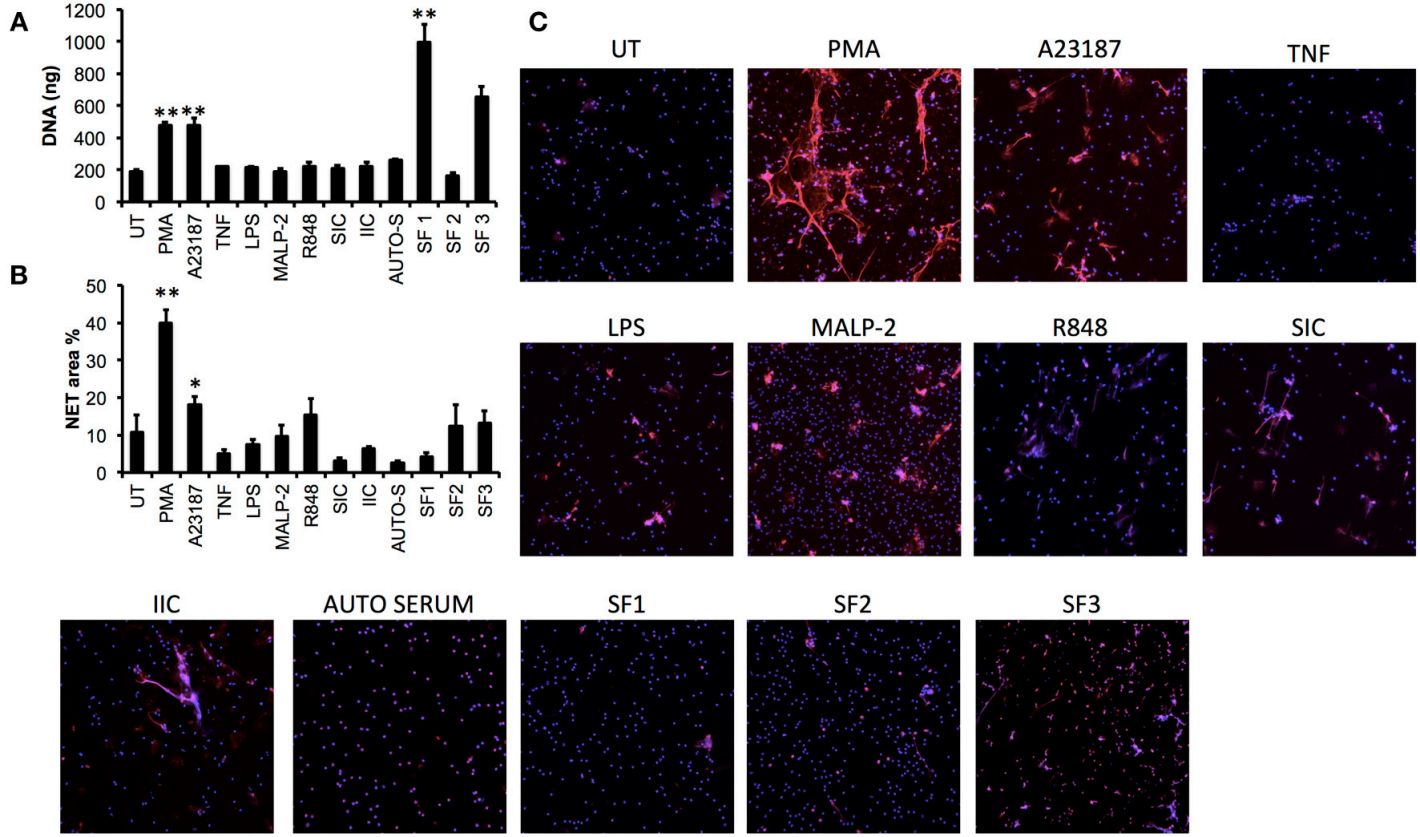
Screening of NET agonists in neutrophils from patients with rheumatoid arthritis (RA). RA neutrophils (*n* = 3) were stimulated with PMA, A23187, TNF, LPS, MALP-2, R848, SIC, IIC, autologous serum (2%, AUTO-S), or RA synovial fluid (SF, *n* = 3 donors). NET production was measured using **(A)** quantification of DNA in culture supernatant following micrococcal nuclease digestion of DNA, and **(B)** quantification of DNA staining (DAPI) using Illastik machine-learning algorithm (^*^p < 0.05, ^**^p < 0.01 compared to untreated). **(C)** Representative immunofluorescent images from *n* = 3 experiments. Images taken on an Epifluorescent microscope (Zeiss) at X20.

In order to determine whether more physiologically-relevant agonists could induce NET production in neutrophils from patients with inflammatory disease, we screened a range of agonists [including TLR- and FcγR-agonists and RA synovial fluid (SF)] using RA neutrophils (*n* = 3). We found that none of the TLR or FcγR agonists screened (LPS, MALP-2, R848, SIC, IIC) induced significant release of NET DNA into culture supernatants measured by micrococcal nuclease assay (Figure 4A), and whilst some MPO-positive NETs were visible by immunofluorescence in response to MALP-2 and R848, these were not statistically significant compared to untreated NET production when analyzed by our Ilastik algorithm (Figures 4B,C). RA SF induced the release of NETs, although this was highly donor dependent both in terms of donor SF (*n* = 3) and donor neutrophils (Figure 4A, *p* < 0.01). TNFα and autologous serum did not induce NETs in RA neutrophils. We did not detect significant levels of spontaneous NETosis in RA neutrophils (Figure 4C), in line with our previous observations (14). The most consistent stimulators of NETs in RA neutrophils were PMA and A23187, which produced significant amounts of NETs in all donors (Figure 4A, PMA 479.9 ± 19.6 ng, A23187 478.0 ± 44.8 ng, *p* < 0.01). NET production in response to A23187 was higher in RA patients compared to healthy controls (Figures 1F, 4A, *p* < 0.01).

For our proteomics experiments using RA and SLE neutrophils (*n* = 6 each), we decided to induce NET production using PMA and A23187, as both these agonists produced consistent NETosis in every healthy and patient donor tested. We used ultra-pure neutrophils in all our experiments; this was confirmed by flow cytometric analysis of CD16^+^ cells and morphological assessment of cytospins (Supplementary Figure 3). We noted that CD16 staining was lower on some SLE neutrophils, with 3/6 patients having two populations of neutrophils: CD16^bright^ and CD16^dim^. We did not observe any eosinophil contamination in these ultra-pure neutrophil preparations and believe the lower CD16 levels on SLE neutrophils may be an early marker of increased apoptosis, in line with previous reports (80).

**Figure 5:**
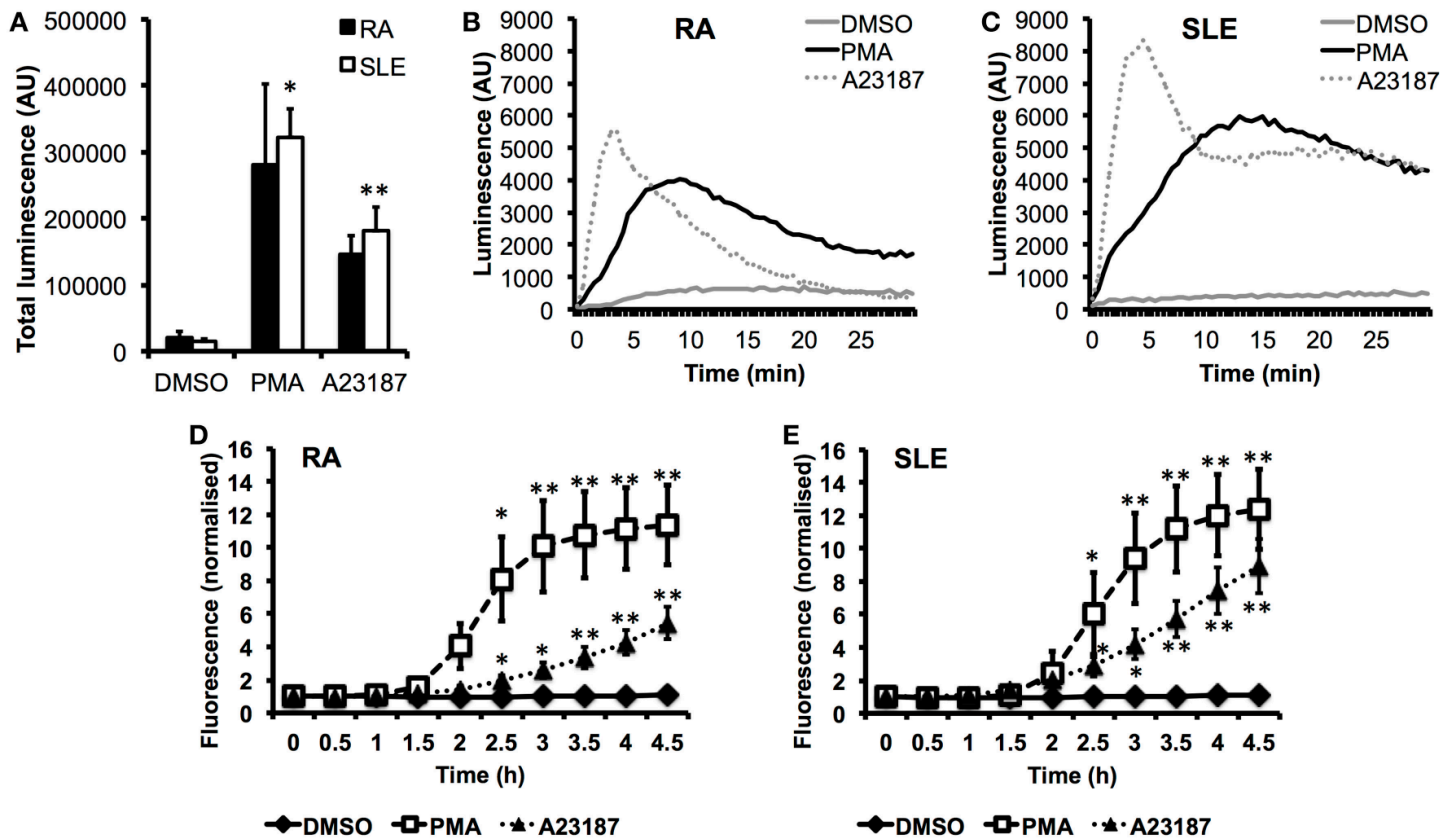
Production of ROS and externalization of DNA by RA and SLE neutrophils in response to PMA and A23187. **(A)** Total ROS production (area under the curve) over 30 min measured by luminol-enhanced chemiluminescence (*n* = 6 each RA and SLE, ^*^p < 0.05, ^**^p < 0.01). Representative traces are shown for RA neutrophils **(B)** and SLE neutrophils **(C)** (AU = arbitrary units). Externalization of DNA measured by SYTOX green assay by RA **(D)** and SLE **(E)** neutrophils (*n* = 6, ^*^p < 0.05, ^**^p < 0.01).

In parallel experiments, we measured ROS production in response to PMA and A23187 (Figures 5A–C). Whilst PMA and A23187 induced significant production of ROS over 30 min by SLE neutrophils (Figure 5A, ^*^p < 0.05, ^**^p < 0.01) the increased levels of ROS produced by RA neutrophils did not reach statistical significance compared to untreated neutrophils (PMA *p* = 0.06, A23187 *p* = 0.31). We additionally noted that ROS production by RA neutrophils peaked around 1.5–2 min sooner than SLE neutrophils in response to both agonists (Figures 5B,C). This suggests that RA neutrophils have been “primed” and released ROS *in vivo* prior to isolation, in line with previous observations (33, 81). We measured the kinetics of NET production by RA and SLE neutrophils in response to PMA and A23187 using the SYTOX green assay. Significant release of NET DNA was observed by 2.5 h with both agonists (Figures 5D,E, ^*^p < 0.05, ^**^p < 0.01). We noted that A23187 induced higher levels of DNA release by SLE neutrophils compared to RA neutrophils, although this did not reach statistical significance (4 h *p* = 0.07). NET production in response to A23187 also occurred faster and was significant by 2 h in SLE neutrophils (*p* = 0.04) but not RA neutrophils (*p* = 0.09). We also noted that the absence or presence of serum greatly affected the amount of NET material produced in response to A23187, but not PMA. The amount of NET material produced in response to A23187 was lower in the presence of 2% serum, likely due to the presence of calcium-binding proteins and/or anti-oxidants in the serum (Supplementary Figure 4).

**Figure 6:**
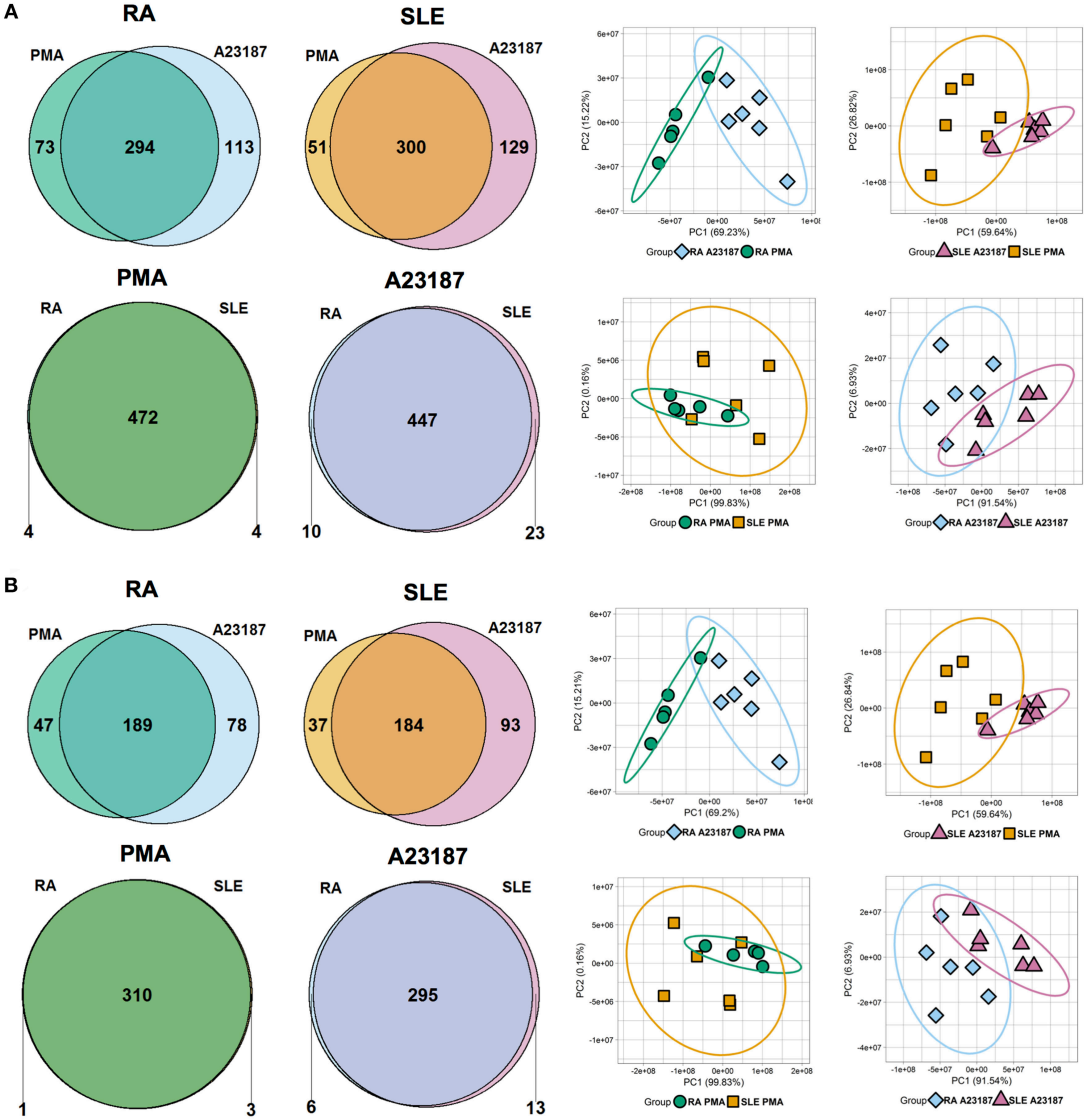
Analysis of NET proteins produced by RA and SLE neutrophils in response to PMA and A23187. Venn diagrams show number of proteins above and below the FDR = 0.05 cut-off for each comparison. Principal component analysis (PCA) shows separation of samples based on significant proteins. Data are shown before **(A)** and after **(B)** filtering for *n* ≥ 2 peptides.

Quantitative proteomics analysis of RA and SLE NETs produced in response to PMA and A23187 identified 480 proteins (Figure 6A, *n* ≥ 1 peptide, Supplementary Table 4). Filtering results to include proteins with *n* ≥ 2 peptides decreased this number to 314 (Figure 6B). The number of proteins significantly different between the four experimental conditions (ANOVA *p* < 0.05) is shown in Figures 6A,B and detailed in Supplementary Table 5. Principal component analysis (PCA) of these proteins separated the samples based on both disease diagnosis and NET agonist, with tight clustering of biological replicates (Figures 6A,B). A number of interesting observations were made in relation to the proteins that were significantly different between the four treatments. Firstly, the identities of the proteins significantly different between PMA and A23187 treatments were similar, independent of whether RA or SLE neutrophils were stimulated, and echoed our observations in healthy control NETs. In general, PMA-induced NETs were decorated with proteins of the annexin family (ANXA1, ANXA4, ANXA5, ANXA6, ANXA7, ANXA11), azurocidin (AZU1/CAP7), and histone H3, whereas A23187-induced NETs were decorated with granule proteins such as CAMP/LL37, CRISP3, lipocalin (LCN2) and MMP8, histones H1.0, H1.4 and H1.5, interleukin-8 (CXCL8), PADI4, and α-enolase (ENO1).

**Figure 7:**
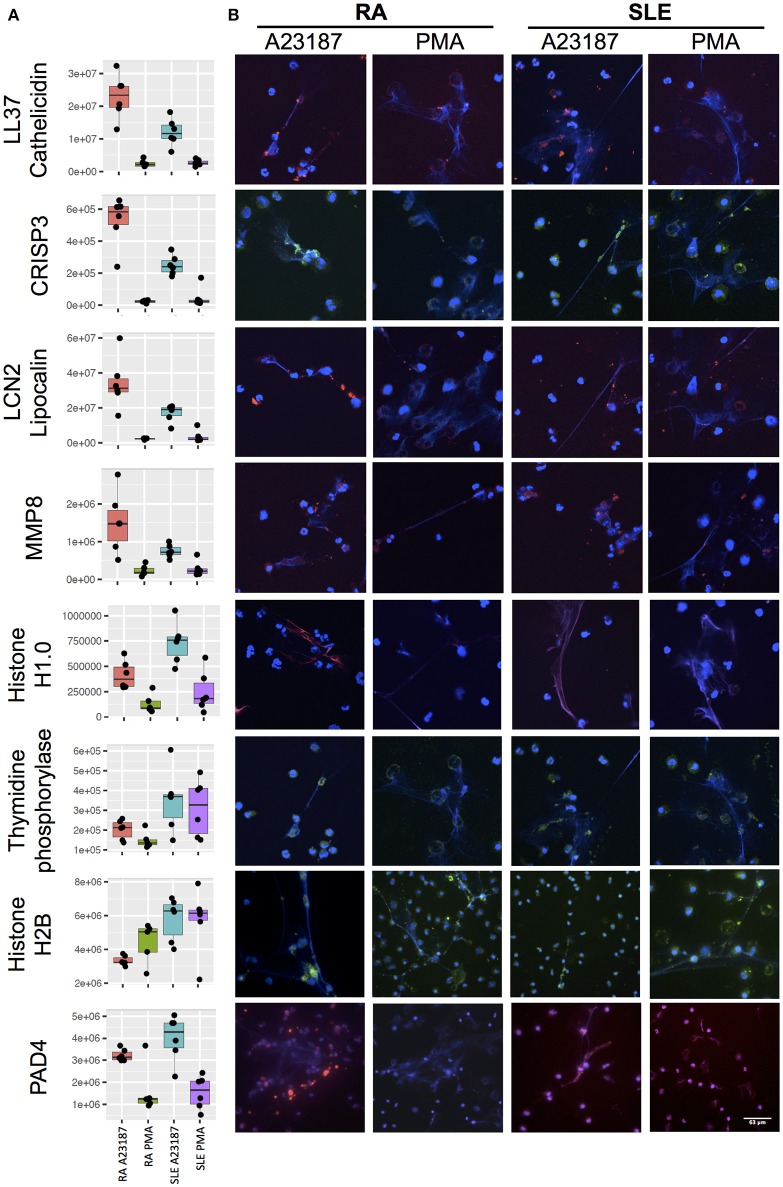
Immunofluorescent staining of NET proteins in RA and SLE NETs produced in response to A23187 or PMA. **(A)** Proteomics data is shown as box-and-whisker plots alongside **(B)** representative immunofluorescent images. Box-and-whisker plots also indicate the median (horizontal line) with dots representing each individual NET proteomics sample. DNA is stained with DAPI (blue), proteins are stained with antibodies as indicated on the figure (LL37/Cathelicidin, LCN2/Lipocalin, MMP8, Histone H1.0, PAD4 stained with AlexaFluor 647 and shown in red; CRISP3, Thymidine phosphorylase, Histone H2B stained with AlexaFluor 488 and shown in green). Images taken on a confocal laser-scanning microscope (Leica DM2500) at X20.

Secondly, a small number of significant differences in NET proteins were identified. When we compared NETs from RA and SLE patient neutrophils stimulated with PMA, four NET proteins were significantly different (Figure 6B, Supplementary Table 5). Non-secretory ribonuclease (RNASE2) was higher in RA neutrophils, whereas MPO, leukocyte elastase inhibitor (SERPINB1) and thymidine phosphorylase (TYMP) were higher in SLE neutrophils. In contrast, when we compared NETs from RA and SLE neutrophils stimulated with A23187, six NET proteins were higher in RA NETs, including CAMP, CRISP3, CXCL8, and MMP8, and 13 NET proteins were higher in SLE NETs, including histones H1.0, H2B (type 1-J), H2B (type 2-F), and H4 (Supplementary Table 5). The presence of a number of these differently expressed NET proteins was confirmed using immunofluorescent staining of NETs prepared and fixed on coverslips in parallel experiments from the same donors. Figure 7 shows the proteomics data (Figure 7A) alongside representative images of antibody-labeled NETs (Figure 7B). The abundance of SERPIN1B on PMA-induced NETs from RA patients correlated with RA disease activity (DAS28) scores (*R*^2^ = 0.84, *p* = 0.029). DAS28 also correlated significantly with phosphoglycerate kinase-1 (PGK1, *R*^2^ = 0.74, *p* = 0.028) and histone H1 family member 0 (H1F0, *R*^2^ = 0.68, *p* = 0.044) in A23187-induced RA NETs.

### Detection of Post-translational Modifications

Several studies have reported the presence of post-translational modifications (PTMs), including citrullination, methylation and acetylation, on NET proteins from RA or SLE patients (47, 49, 55, 82). These PTMs may play a key role in the development of auto-immunity, for example the production of auto-antibodies to citrullinated peptides in RA and acetylated histones in SLE. Moreover, serum from RA and SLE patients cross-reacts with NET proteins containing PTMs (47, 83). We carried out analysis of PTMs in our proteomics data using the software PEAKS. Acetylation and methylation modifications were selected using standard PEAKS search terms, and citrullination (not a standard search term) was identified using a missed arginine cleavage together with an increase in MW of 0.98Da (84). All PTMs called by PEAKS were manually inspected for inclusion in the results, for example if a citrullination event was predicted in the C-terminal position of a peptide (i.e., a trypsin cleavage site) this was eliminated as an incorrect identification.

We initially searched for citrullinated peptides in the healthy control samples. PEAKS analysis identified two peptides with citrulline modifications in all three samples of A23187-induced NETs from histone H1.5, H1.3, or H1.4 and glial fibrilary acidic protein (Supplementary Table 6A). Four proteins were identified as having citrulline modifications in all three samples of PMA-induced NETs: azurocidin, neutrophil elastase, myeloid cell nuclear differentiation antigen (MNDA), and histidine decarboxylase (Supplementary Table 6A). Several other citrullinated peptides were identified but were not found to contain citrulline modifications in all samples (Supplementary Table 6A); for example citrullinated histone H3.1t peptide was found in 3/6 samples (two PMA-induced and one A23187-induced sample).

PEAKS identified histone H3 as the only citrullinated peptide all RA and SLE patient samples (Supplementary Table 6B). It is noteworthy that citrullinated histone H3 was present in all RA and SLE samples, but was only present in 3/6 healthy control samples, possibly indicating *in vivo* citrullination of histone H3 in disease. Eighteen peptides (from 11 proteins) were citrullinated in >50% of NET samples (irrespective of agonist), including MNDA, vimentin (VIM), actin, high mobility group protein B2 (HMGB2), non-histone chromosomal protein HMG-17 (HMGN2), lamin-B receptor (LBR), and histone H1.3/H1.4/H1.5 (Supplementary Table 6B). Nineteen peptides (from 11 proteins) were citrullinated in >50% A23187-induced NETs (6/12 samples), but <50% PMA-induced NETs (5/11 samples) (Supplementary Table 6C). These included histone H1.3/H1.4/H1.5, and HMGB1/2, as well as myosin 9/10/14. Nine peptides (from eight proteins) were citrullinated in >50% PMA-induced NETs, but <50% A23187-induced NET samples (Supplementary Table 6D). These included lactotransferrin and MPO. We did not observe any difference in citrullinated peptides between the RA and SLE samples.

In total, eighteen peptides (from 13 proteins) had acetylation modifications in at least 22/23 samples, including histones 1.0 1.2, 1.3, and 1.5, actin (cytoplasmic 1 or 2), thymosin beta-4 and annexin A1 and A3 (Supplementary Table 6E). Four thymosin beta peptides had the same acetylation site, but different trypsin cleavage profiles or other additional PTMs. Thirty-one peptides were acetylated in >50% A23187-induced NET samples but <50% PMA-induced NET samples (Supplementary Table 6F). Many of these were from histones, in particular histone H3 isoforms. Eleven peptides were acetylated in >50% PMA-induced NETs but <50% A23187-induced NETs (Supplementary Table 6G). Of note, we detected acetylated peptides from histone H2B and histone H4 in A23187-induced NETs but not in PMA-induced NETs. This could be because the acetylated tails of histone H2B are cleaved during NOX-dependent (i.e., PMA-induced) NETosis, as has been reported previously (55).

Methylation events were identified in three peptides (from two proteins) in at least 22/23 samples: actin (cytoplasmic 1 or 2) and histone H3.2 (Supplementary Table 6H). Nine methylated peptides were present in >50% A23187 samples and <50% PMA samples (Supplementary Table 6I) and four methylated peptides were present in >50% PMA samples and <50% A23187 samples (Supplementary Table 6J). The majority of the former were peptides from histone H3.1, H3.2, and H3.3.

**Figure 8:**
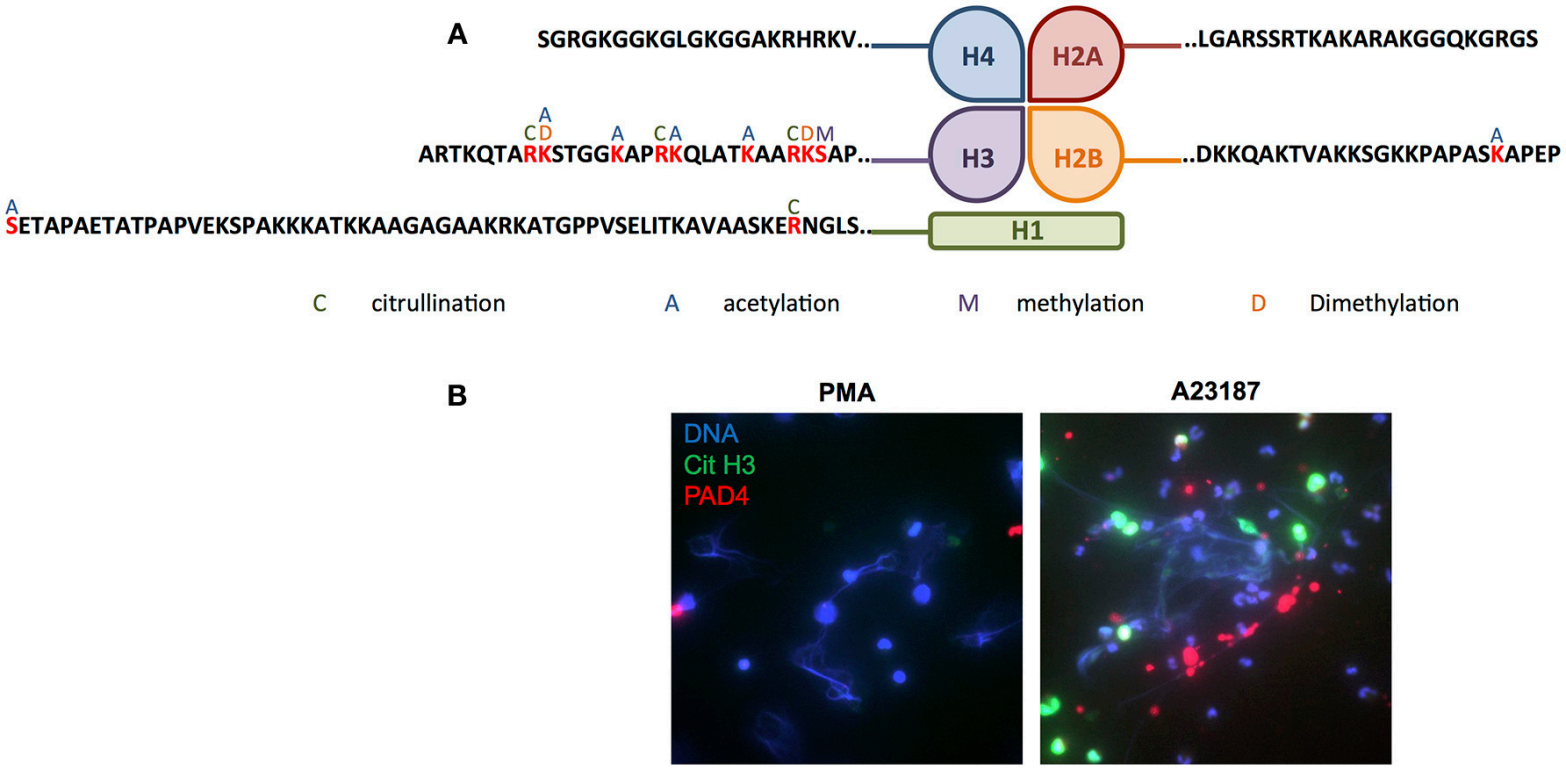
Post-translational modification of histones in NETs. **(A)** Summary of the post-translational modifications to the N-terminal tails of histones H1, H2A, H2B, H3, and H4 in RA and SLE neutrophil NETs (C, citrullination; A, acetylation; M, methylation; D, dimethylation). **(B)** Representative immunofluorescent staining of DNA (blue), citrullinated histone H3 (green) and PADI4 (red) in PMA- and A23187-induced NETs. Images taken on an Epifluorescent microscope (Zeiss).

Several proteins, in particular members of the histone family (histones H1.3/H1.4/H1.5, H2B, H3.1, and H3.3), had multiple PTMs including citrullinated, acetylated and methylated protein residues. Modifications of the N-terminal tails of histones detected in our study are summarized in Figure 8A. Immunofluorescent staining of citrullinated histone H3, and the presence of PADI4 on A23187-induced NETs, is shown in Figure 8B. A full list of all peptides with PTMs identified across all RA and SLE samples can be found in Supplementary Table 7. Example spectra from peptides with citrullination, acetylation and methylation modifications can be found in Supplementary Figures 5–7, respectively.

## Discussion

In this study we have applied quantitative proteomics to investigate the protein content of NETs generated by healthy control, RA and SLE neutrophils in response to PMA (NOX2-dependent NETosis) and A23187 (NOX2-independent NETosis). We have identified significant differences in NET proteins released in response to these two agonists. We have also directly compared NETs from RA and SLE neutrophils, and show there are only a small number of proteins that are significantly different. In addition we have described a number of peptides containing citrullination, acetylation and/or methylation PTMs.

As part of this study we screened a wide-range of agonists, including TLR and FcγR agonists, for their ability to induce NETosis in RA neutrophils. Of note, LPS did not induce NETosis in our experimental system. This may be due to our use of ultra-pure neutrophils [important for the proteomics aspect of this study (85)], as LPS-induced NETosis has been shown to be critically dependent upon the presence of platelets (86, 87). We also noted lower production of NETs in response to A23187 than reported in other studies (16, 17), likely due to our inclusion of serum (containing calcium-binding proteins and anti-oxidants). In general we found that SLE neutrophils were more responsive to A23187-stimulation, both in terms of ROS production and NET release. We believe there could be two possible explanations for this. Firstly, RA peripheral blood neutrophils are primed *in vivo* and thus may have released ROS prior to isolation (33, 81). Secondly, the SLE patients in our study were significantly younger than the RA patients. Several neutrophil functions, including the production of ROS and NETosis, decline with age (88).

### NET Proteins Implicated in SLE Pathogenesis

More than 100 different auto-antibodies have been identified in SLE (89). Antibodies against histones are particularly common, including auto-antibodies against histone H1, H2A, H2B, H2A-H2B, H3, and H4 (90–95). HMG-17 auto-antibodies correlate with both SLE disease activity and anti-dsDNA auto-antibody titres (96). Auto-antibodies that recognize catalase (97), lamin B1 and B2 (98–101), apolipoprotein A1 (102), annexin AI and α-enolase (103–105) have also been described in SLE, with annexin AI and α-enolase auto-antibodies being associated with skin and kidney involvement (104, 105). All of the aforementioned proteins were identified in SLE NETs our study.

MMP9 in SLE LDG NETs impairs aortic endothelium-dependent vasorelaxation and induces endothelial cell apoptosis (59). Cathelicidin (CAMP/LL37) has a strong association with the onset of SLE (106) and is elevated in both serum and skin of SLE patients (107, 108). However, CAMP/LL37 auto-antibody titres do not correlate with SLE disease activity (108). Interestingly we observed higher levels of CAMP/LL37 in RA NETs than we did in SLE NETs.

### NET Proteins Implicated in RA Pathogenesis

Many of the NET proteins identified in our analysis are elevated in RA synovial fluid, including cathepsins, MPO, MMP8, MMP9, LCN2, and PADI2 (109–113). These proteins contribute to cellular infiltration and degradation of collagen within synovial joints (114). Elevated serum MMP8 is a strong predictor of mortality in RA (115). RNASE2, elevated in RA NETs, is a potential gene biomarker of TNFi-refractory RA (67).

Patients with RA and Felty's syndrome (a complication of RA) also have auto-antibodies to histones (116, 117), with auto-antibodies in sera from patients with Felty's syndrome cross-reacting with citrullinated histones bound to NETs (49). RA sera, particularly those with high ACPA titres, stimulate NETosis and cross-react with citrullinated histone H4 derived from neutrophil NETs (50). Citrullinated histone H4 was previously identified in NETs (50), although we did not detect this in our study. B cell clones isolated from RA synovial tissue produce antibodies which strongly react with citrullinated forms of histones H2A/H2B, fibrinogen and vimentin (47). These antibodies also cross-react with NETs produced *in vitro* in response to PMA (47). Citrullinated histone H2B is arthrogenic in a mouse model of inflammatory arthritis (118) and we identified two citrullinated H2B peptides in some (but not all) RA patient samples.

### Histone Family Proteins in NETs

Major findings in our proteomics data were (i) the significant differences in the externalization of histone family proteins in response to PMA and A23187, (ii) the differences in histone externalization between RA and SLE NETs, and (iii) the range of PTMs on histones. In general, we found that the core histones (H2A, H2B, H3, and H4) were expressed higher on PMA-induced NETs, whereas the linker histones (H1.0, H1.4, and H1.5) were expressed higher on A23187-induced NETs. We also observed fewer histone PTMs in response to PMA than A23187. Both these phenomenon are likely due to the activity of elastase which both rapidly degrades the linker histones (23) and cleaves the N-terminus of core histones during NOX2-dependent NETosis (55).

Histones H2B and H4 are major auto-antigens in SLE (119, 120), and interestingly we found these were significantly higher in A23187-induced NETs from SLE patients. Auto-antibodies against a number of histone PTMs have been found in SLE (8, 48, 120, 121). For example, acetylated histone H2B (K12/K20), citrullinated histone H3 and acetylated histone H4 are recognized by SLE serum auto-antibodies (8, 48), although the PTMs identified in our study were on different lysine residues from this earlier study. We did however, detect methylation of histone H3 lysine-27 (H3K27), a known auto-antigen in SLE (121).

Histone acetylation has been suggested to enhance the immuno-stimulatory potential of NETs in SLE (122). A recent study showed that histones from unstimulated SLE neutrophils are hypoacetylated and hypomethylated when compared to neutrophils from healthy donors. However, NETs from SLE patients were found to contain higher amounts of acetylated histone H4-K8, K12, K16, acetylated histone H2B-K12 and tri-methylated histone H3-K27 (123). Our study identified histone H4 acetylated at K8, K12, and K16. We also found acetylation of histone H4K5, which had previously been identified in NETs generated in response to H_2_O_2_ (8).

### Citrullinated NET Proteins

A number of proteins identified on NETs in our analysis contained one or more sites of citrullination. Again a high proportion of these proteins were histones (both core and linker histones), as well as actin, vimentin, MNDA, and HMGB2. Many citrullinated host proteins act as sources of autoantigens in RA; for example citrulline residues in aggrecan and vimentin are preferentially recognized by antigen-presenting cells in individuals carrying the HLA-DRB1^*^04:01/04 allele (which contains the “shared susceptibility epitope”) (124). A combination of the presence of ACPA auto-antibodies and the presence of the HLA-DRB1^*^04:01/04 allele is strongly associated with the development of RA (125). Citrullinated vimentin is the antigenic target of anti-Sa auto-antibodies, and is present in the sera and synovial tissue of RA patients (126). NETs containing citrullinated peptides are internalized by synovial fibroblasts via a RAGE-TLR9 pathway, and then presented via MHC Class II to antigen-specific T-cells (83). HLA-DRB1^*^04:01 transgenic mice develop auto-antibodies specific to citrullinated forms of NET peptides, including core histones (H2A, H2B, H3), α-enolase and vimentin (83).

A recent study suggested RA NETs produced in response to rheumatoid factor contain citrullinated azurocidin, catalase, histone H2A (type 2C), histone 2B, MPO, elastase, profilaggrin, protein S100-A12, and protein S100-A9 (83). However, many of the citrulline peptides identified in this recent study (83) were reported at the C-terminus of the peptide (trypsin cleavage site). As citrulline does not have the positive charge of arginine, the general assumption is that citrullination will result in a missed cleavage by trypsin (84), and therefore the assignment of these peptides is questionable. Indeed, we excluded any peptides with a reported C-terminal citrulline from our own analysis.

Recent analysis of the RA synovial citrullinome showed wide-spread protein citrullination within RA synovial fluid, and identified a number of citrullinated proteins that correspond with our dataset, such as actin (beta and gamma), cathepsin G, coronin, gelsolin, histone H1.3, histone H3.3, MNDA, MPO, myosin 9, and vimentin (110). Several of the PTMs identified corresponded to the arginine residue detected in our dataset, suggesting that NET products and/or NET-derived PAD enzymes may be responsible for the presence of these modified proteins (127, 128). Citrullination sites on histone H1 family proteins induced by PADI4 activity in response to A23187 have been detailed previously, and correspond to some of the citrulline PTMs identified in our dataset (82).

We detected both PADI4 and PADI2 in our datasets, although PADI2 was not significantly different between treatment conditions. PADI2 is the key enzyme involved in TNFα-induced protein citrullination in mouse models of RA (129). In this model, PADI4 did not induce citrullination of proteins in mouse ankles, but was essential for NET formation, and only PADI2 knockout improved clinical measurements of disease activity (129). RA patients with severe, erosive disease have auto-antibodies to PADI3/PADI4 which can increase the catalytic activity of PADI4 in a forward-feedback loop associated with high levels of disease activity (130). Indeed, PADI4 has emerged as a potential therapeutic target for the treatment of RA and has shown efficacy in some, but not all, PADI4 knock-out models of inflammatory arthritis (129, 131, 132).

It was recently shown that citrullinated histones, particularly citrullinated histone H3, mediate microvascular leakage, and endothelial barrier dysfunction (133). Though it is not entirely clear whether the same results would have been obtained with unmodified histones, this recent work (133) demonstrates the concept that PTM NET products are able to affect the physiology of other cells as well as being major auto-antigens. Citrullination of peptides in NETs may also alter their function, for example CAMP/LL37 can be citrullinated at three or five sites; CAMP/LL37 citrullinated at 5 residues is the most chemotactic to PBMCs and most pro-inflammatory (134).

### Future Challenges for the Field

We believe that our use of a global approach to NET proteomics rather than a hypothesis-driven, targeted approach highlights the fact that, with only a few exceptions, NET proteins are broadly similar irrespective of health or disease, and that the differences lie in the stimulus and mode of NET production (NOX2-dependent or NOX2-independent). Indeed, we believe that previous, hypothesis-driven research may have missed this important point. For example, there are a number of publications on the importance of NET-derived CAMP/LL37 in SLE and on the involvement of PADI4 activity in RA (45, 106–108, 130). However, we have shown that both CAMP/LL37 and PADI4 are presented on both RA and SLE NETs depending upon the agonist used. This raises two possibilities: first, that the stimuli leading to NET production *in vivo* in the respective diseases are different, or second it is the response of the adaptive immune system to the NET material that is the driver of disease manifestation. Fully understanding the drivers of NETosis *in vivo* is more important than ever. We agree with many others in the field that there are two different processes that are both often confusingly referred to as NETosis. Several groups are now making the distinction between NOX2-dependent and NOX2-independent NETosis (17, 25, 55). A recent publication even suggested referring to NOX2-independent NETosis as leukotrophic hypercitrullination (135).

Further subtleties in the signaling of physiological stimuli of NOX2-dependent and NOX2-independent NETosis may exist that are not captured by merely using PMA and A23187. We further echo the desire of others (136) to see the field become more standardized in the experimental approaches used decipher NETosis, including more quantitative and high through methodologies. We believe, that we have described both a high throughput proteomic and high throughput imaging technique. Analysis of NET remnants in serum of patients with RA and SLE suggests NETs produced *in vivo* in both diseases originate via NOX2-independent NETosis (55). Therefore, perhaps PMA is a poor model for future *in vitro* studies seeking to replicate NETosis in inflammatory disease.

The challenge in the field is to design experiments that recapitulate conditions *in vivo* e.g., inclusion of platelets, endothelial cells, and other immune cells, the use of biofluids such as sera and synovial fluid as agonists, and the measurement of NET proteins in tissue biopsies. However, this poses significant challenges particularly for proteomics studies, i.e., isolation of neutrophil and NET proteins from mixed-cell populations, and carry-through of proteins from sera and synovial fluid into proteomic analyses.

The importance of NETs and NET proteins has wide-reaching implications in medicine. It was recently shown that NET fragments promote innate immune responses that prevent lung transplant tolerance (137), and that NETs cause dendritic cell maturation and subsequent Th1 cell expansion (138). We must understand NETs further if we wish to suppress these unwanted immune responses. Future studies might also examine whether medication affects the protein contents of NETs, as suggested by a recent study showing clarithromycin increased CAMP/LL37 load on NETs (139).

### Final Summary

This work provides the first, direct comparison of NOX2-dependent (PMA) and NOX2-independent (A23187) NETs using quantitative proteomics. We have also undertaken the first direct comparison of NETs from RA and SLE using proteomics, and show that whilst there are a small number of proteins that are significantly different between RA and SLE NETs, for example histone H2B is higher in SLE, it is the nature of the stimulant rather than the underlying neutrophil physiology that determines the NET protein patterns. We additionally identify an extensive range of post-translationally modified proteins in RA and SLE, many of which are known targets of auto-antibodies in each disease. This work provides important insight into how the regulation of NETosis, and exposure of intracellular antigens on NETs, may contribute to the pathophysiology of RA and SLE.

## Data Availability

The datasets generated for this study can be found in PRIDE (73), doi: 10.6019/PXD011796.

## Ethics Statement

This study was carried out in accordance with the recommendations the University of Liverpool Committee on Research Ethics (CORE) for healthy controls, and National Research Ethics Service (NRES) Committee Northwest (Greater Manchester West) for RA and SLE patients with written informed consent from all subjects. All subjects gave written informed consent in accordance with the Declaration of Helsinki. The protocol was approved by CORE and NRES.

## Author Contributions

EC designed the experiments, carried out the experiments, analyzed the data, and wrote the manuscript. ML carried out the experiments and analyzed the data and reviewed the manuscript. DS carried out the proteomics analysis and revised the manuscript. DM designed the image analysis macro, analyzed the data, and revised the manuscript. RB designed the proteomics experiments and revised the manuscript. RM provided patients and clinical advise, and revised the manuscript. HW designed the study, carried out the experiments, analyzed the data, and wrote the manuscript.

### Conflict of Interest Statement

The authors declare that the research was conducted in the absence of any commercial or financial relationships that could be construed as a potential conflict of interest.
