# Supplementary figures and images for: Caught in a Trap? Proteomic Analysis of Neutrophil Extracellular Traps in Rheumatoid Arthritis and Systemic Lupus Erythematosus
Source: Front Immunol. 2019 Mar 11;10:423. doi: 10.3389/fimmu.2019.00423 (PMC6421309; doi:10.3389/fimmu.2019.00423)

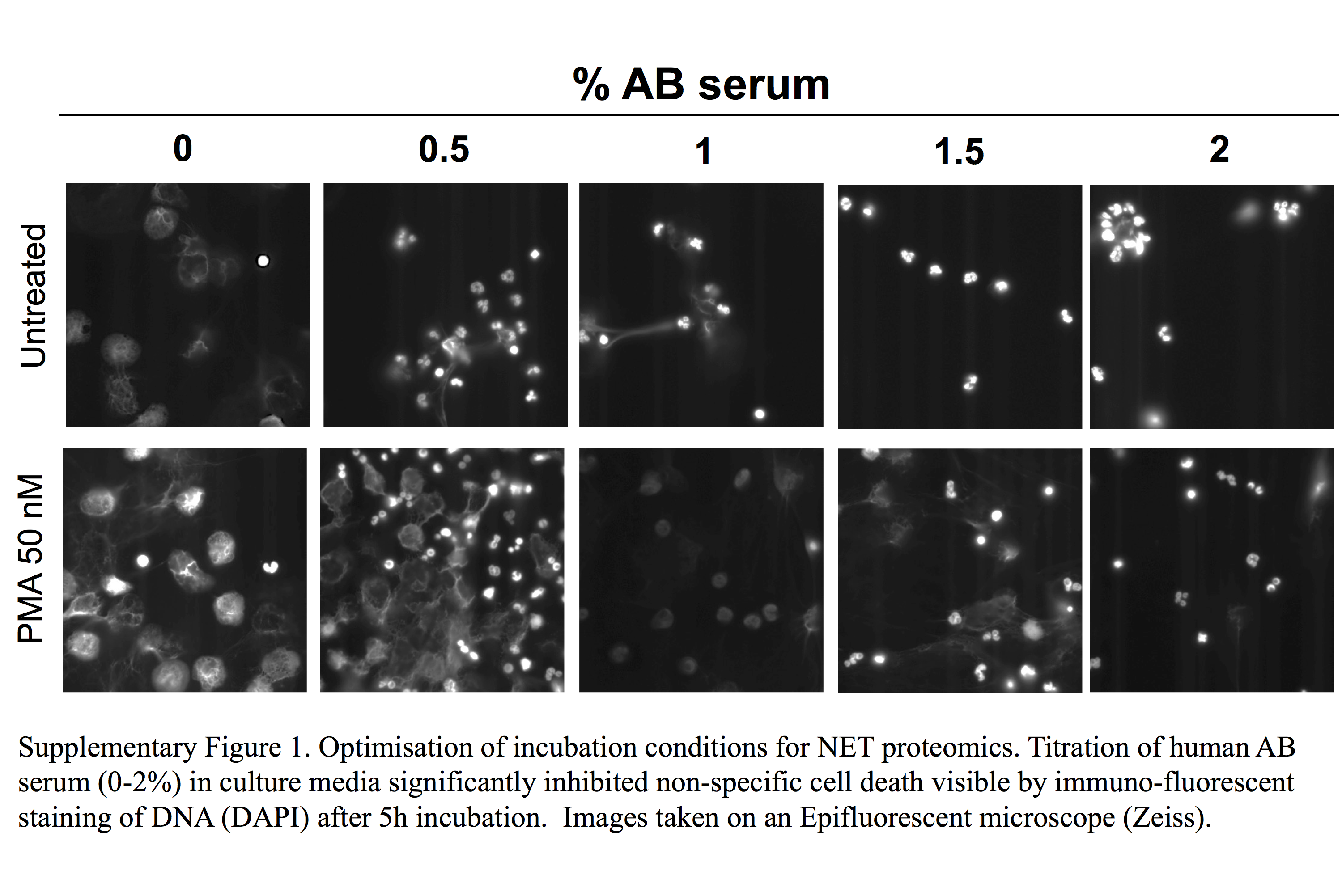

Supplement: Supplementary file 8 [file Image_1.TIFF]

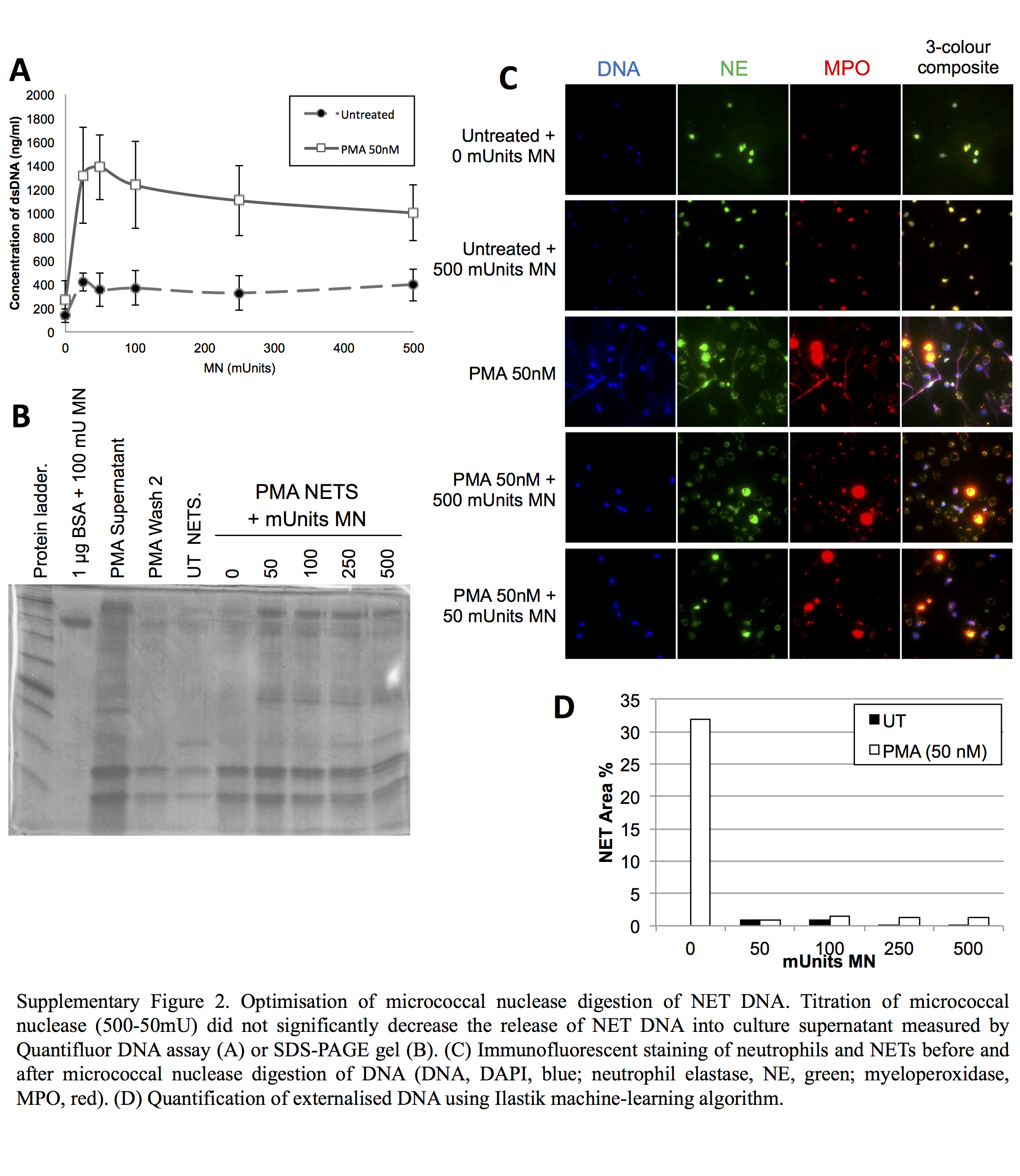

Supplement: Supplementary file 9 [file Image_2.TIFF]

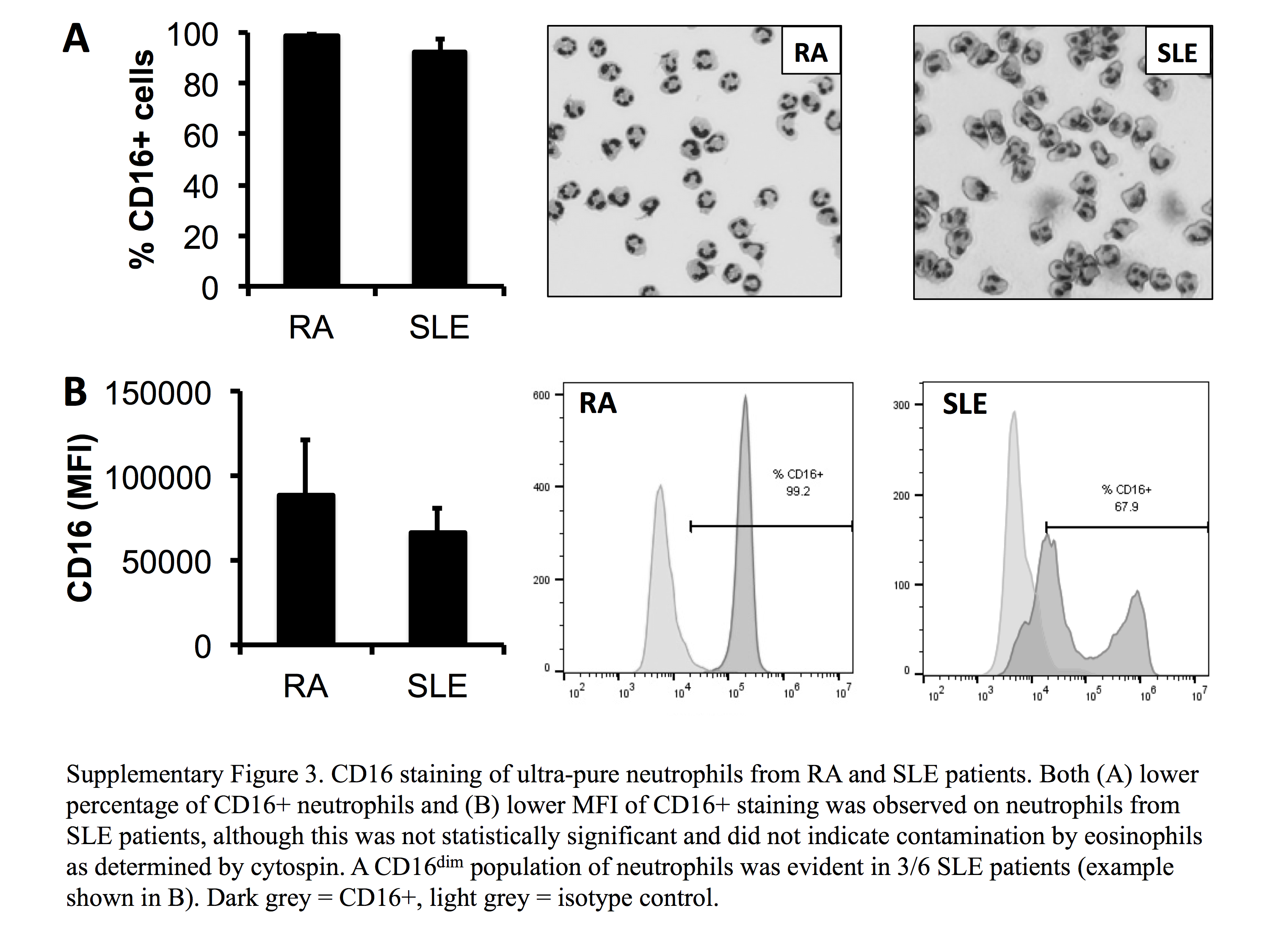

Supplement: Supplementary file 10 [file Image_3.TIFF]

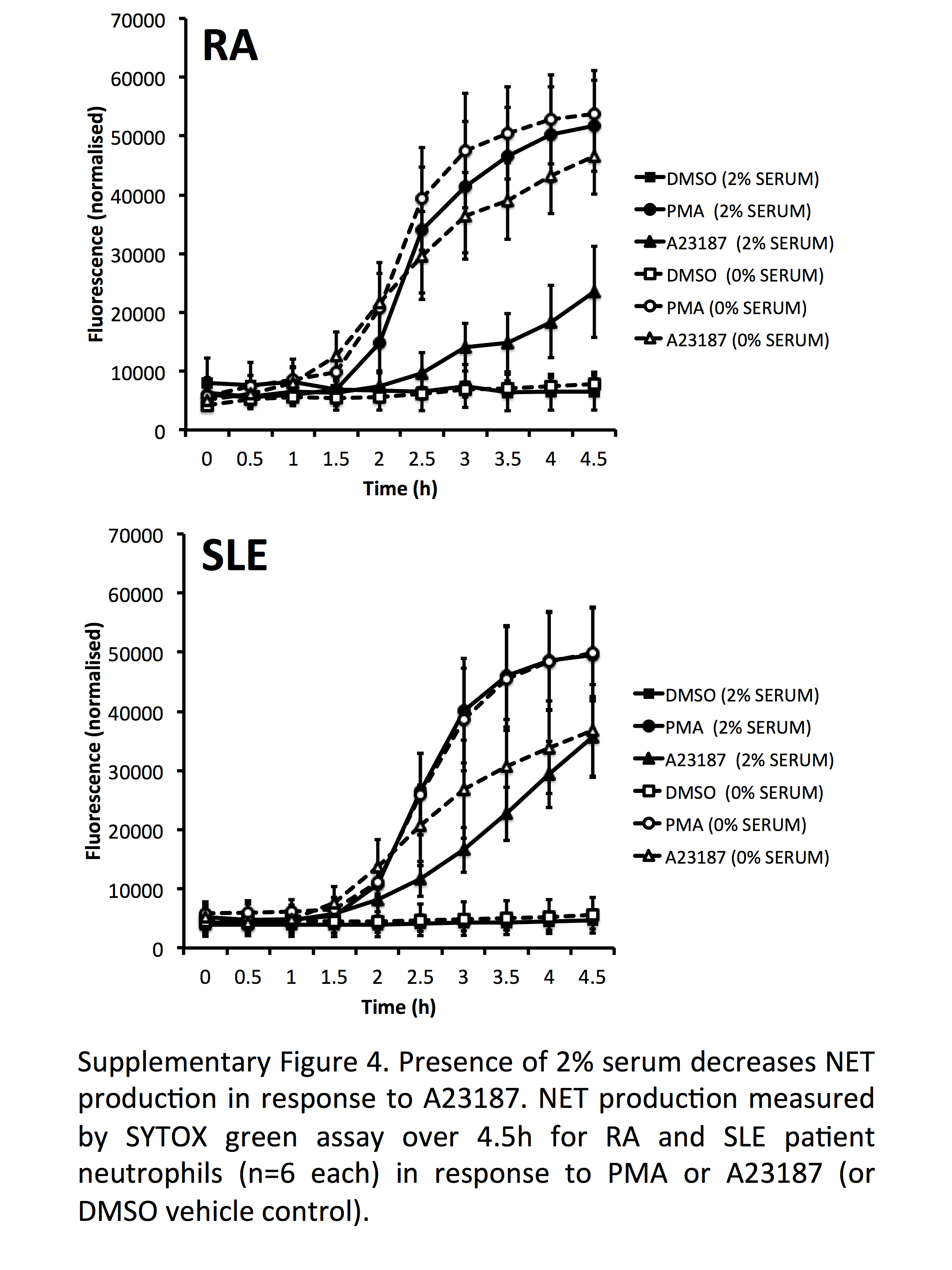

Supplement: Supplementary file 11 [file Image_4.TIFF]

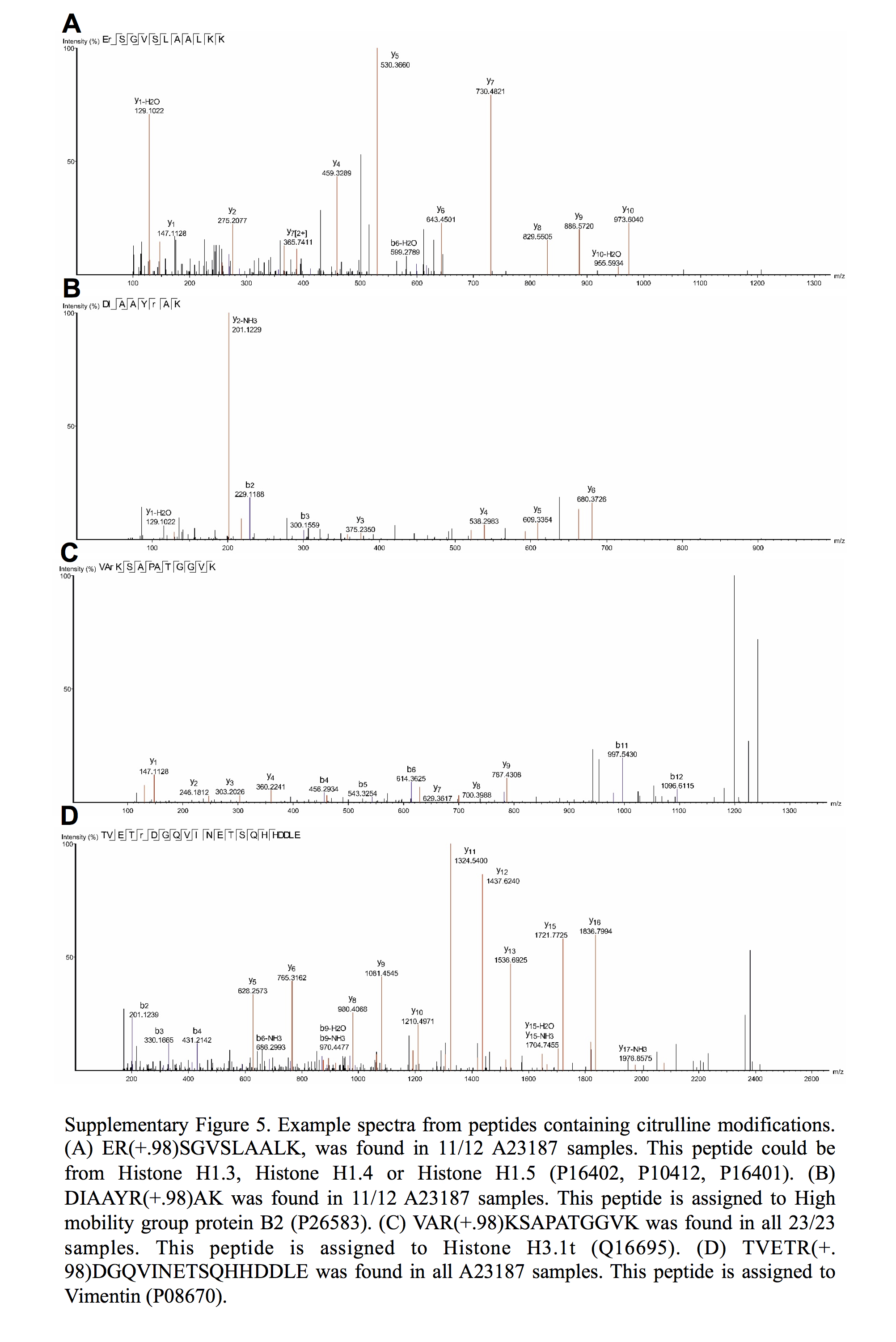

Supplement: Supplementary file 12 [file Image_5.TIFF]

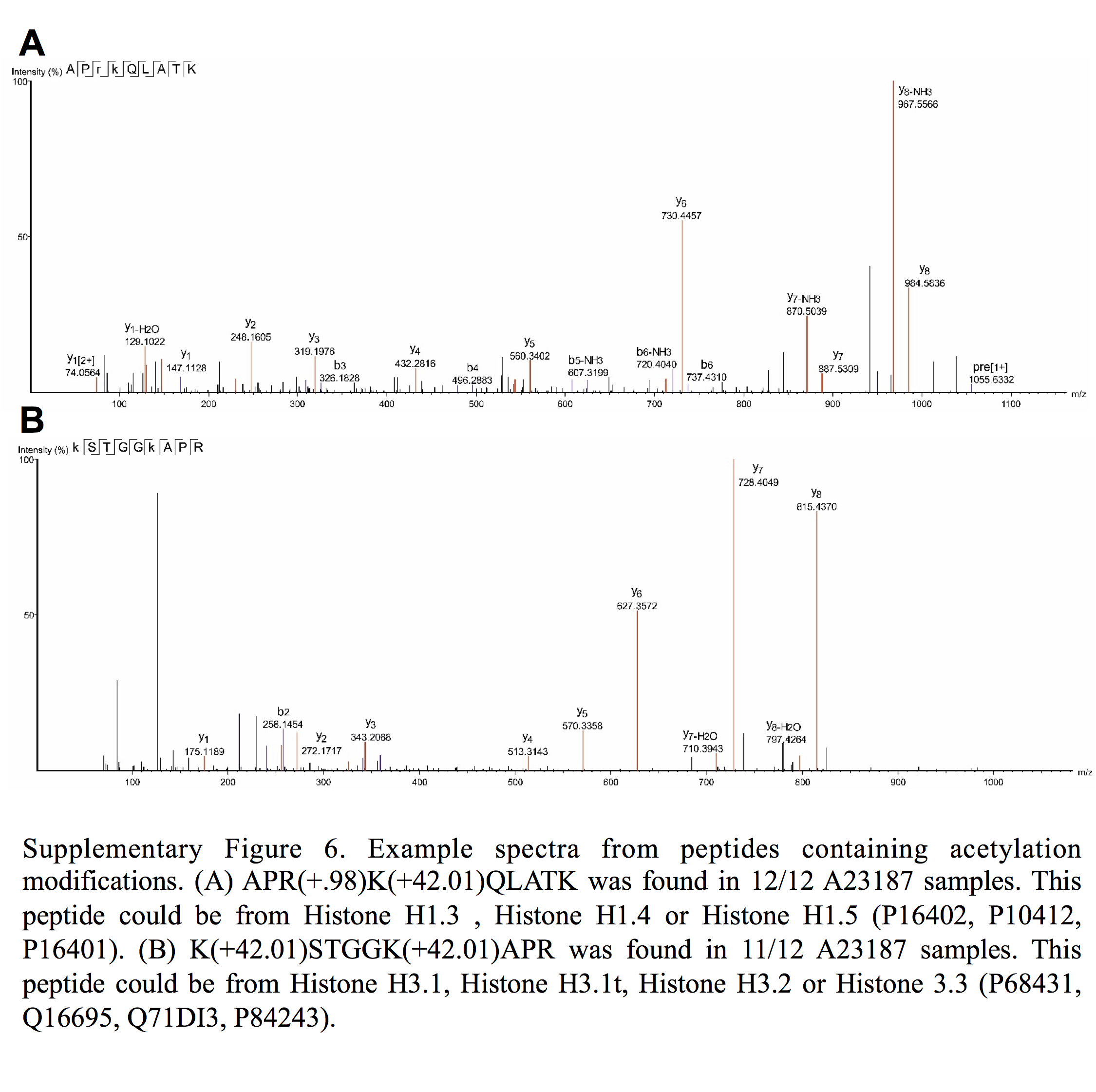

Supplement: Supplementary file 13 [file Image_6.TIFF]

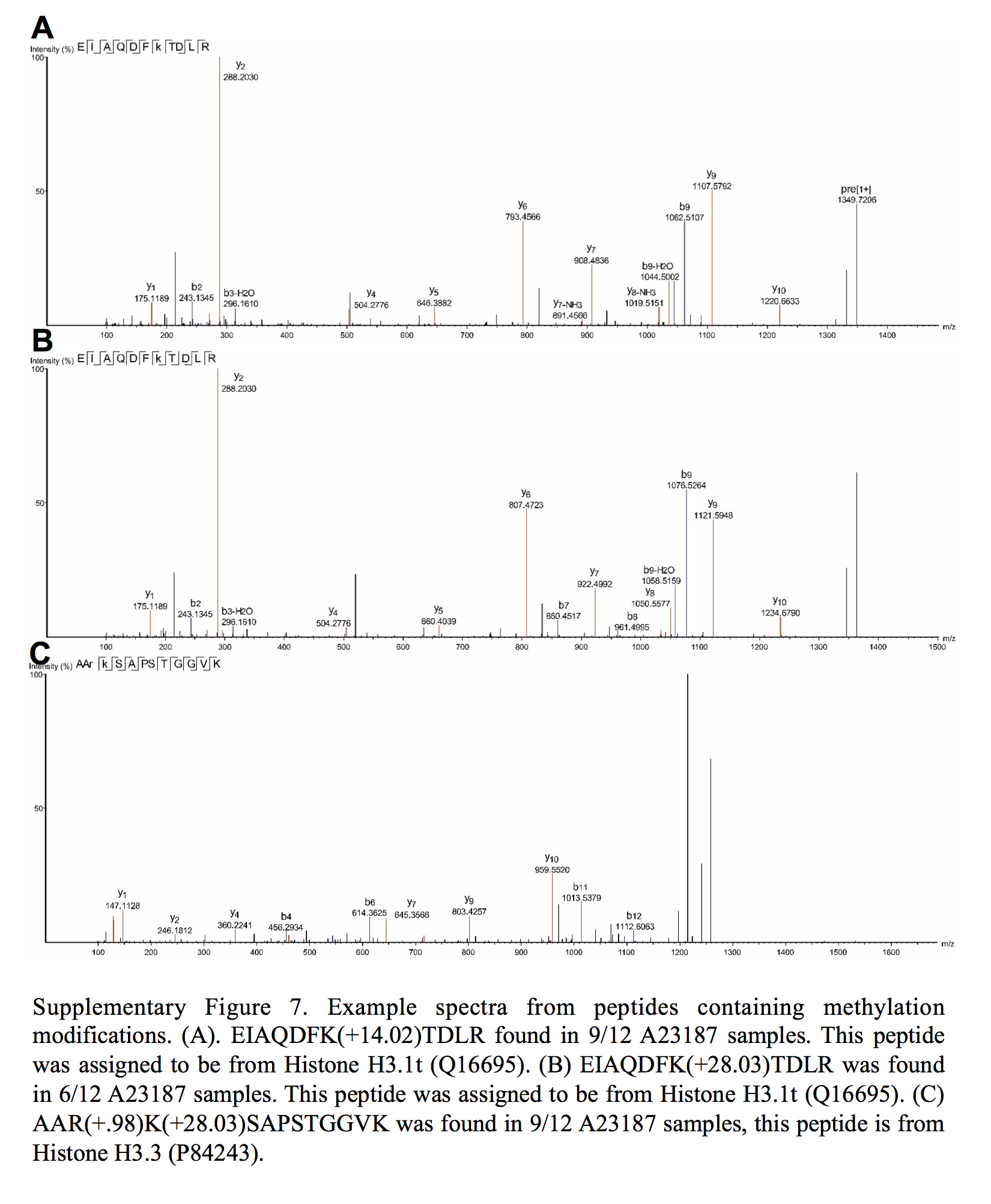

Supplement: Supplementary file 14 [file Image_7.TIFF]
